# Integrative medicine for subacute stroke rehabilitation: a study protocol for a multicentre, randomised, controlled trial

**DOI:** 10.1136/bmjopen-2014-007080

**Published:** 2014-12-04

**Authors:** Jianqiao Fang, Lifang Chen, Luni Chen, Chao Wang, Crystal Lynn Keeler, Ruijie Ma, Shouyu Xu, Laihua Shen, Yehua Bao, Conghua Ji

**Affiliations:** 1 Department of Acupuncture, The Third Affiliated Hospital of Zhejiang Traditional Chinese Medical University, Hangzhou, Zhejiang, China; 2 The Third Clinical Medical College of Zhejiang Chinese Medical University, Hangzhou, Zhejiang, China; 3 Department of Innovations to Wellness, Affiliated with Five Branches University, San Jose, California, USA; 4 Department of Rehabilitation, The Third Affiliated Hospital of Zhejiang Chinese Medical University, Hangzhou, Zhejiang, China; 5 Department of Acupuncture & Encephalopathy, Jiaxing Hospital of Traditional Chinese Medicine, Jiaxing, Zhejiang, China; 6 Department of Acupuncture & Rehabilitation, Hangzhou Hospital of Traditional Chinese Medicine, Hangzhou, Zhejiang, China; 7 The Clinical Research Institute of Zhejiang Provincial Hospital of Traditional Chinese Medicine, Hangzhou, Zhejiang, China

**Keywords:** STROKE MEDICINE, COMPLEMENTARY MEDICINE

## Abstract

**Introduction:**

Many patients with stroke receive integrative medicine in China, which includes the basic treatment of Western medicine and routine rehabilitation, in conjunction with acupuncture and Chinese medicine. The question of whether integrative medicine is efficacious for stroke rehabilitation is still controversial and very little research currently exists on the integrated approach for this condition. Consequently, we will conduct a multicentre, randomised, controlled, assessor-blinded clinical trial to assess the effectiveness of integrative medicine on stroke rehabilitation.

**Methods and analysis:**

360 participants recruited from three large Chinese medical hospitals in Zhejiang Province will be randomly divided into the integrative medicine rehabilitation (IMR) group and the conventional rehabilitation (CR) group in a 1:1 ratio. Participants in the IMR group will receive acupuncture and Chinese herbs in addition to basic Western medicine and rehabilitation treatment. The CR group will not receive acupuncture and Chinese herbal medicine. The assessment data will be collected at baseline, 4 and 8 weeks postrandomisation, and then at 12 weeks’ follow-up. The primary outcome is measured by the Modified Barthel Index. The secondary outcomes are the National Institutes of Health Stroke Scale (NIHSS), Fugl-Meyer Assessment, the mini-mental state examination and Montreal Cognitive, Hamilton's Depression Scale and Self-Rating Depression Scale, and the incidence of adverse events.

**Ethics and dissemination:**

Ethical approval was obtained from ethics committees of three hospitals. The results will be disseminated in a peer-reviewed journal and presented at international congresses. The results will also be disseminated to patients by telephone, during follow-up calls inquiring on patient's post-study health status.

**Trial registration number:**

Chinese Clinical Trial Register: ChiCTR-TRC-12001972, http://www.chictr.org/en/proj/show.aspx?proj=2561

## Introduction

Stroke is the second most common cause of death and leading cause of adult disability worldwide.^1^ The number of patients who die from stroke is more than three times that of those who die from coronary heart disease.^2^ Modern Western medicine in China undoubtedly occupies the dominant position in prevention and treatment of stroke. However, most patients with stroke are treated with one or more types of traditional Chinese medicine (TCM) in addition to Western medicine. The role of TCM should not be ignored.^3–5^ With the development of integrative medicine (IM), which was established in the 1980s, more and more patients with stroke receive IM treatment. The main integrative treatment includes the standard Western medicine and rehabilitation for stroke, as well as acupuncture and (or) Chinese herbs.^6^ Nevertheless, there is not yet enough evidence to show the effect of IM for stroke. More rigorously designed, large scale, multicentre randomised trials are necessary to assess the effectiveness of IM on stroke rehabilitation.^7^

## Methods

### Study design

**Figure 1:**
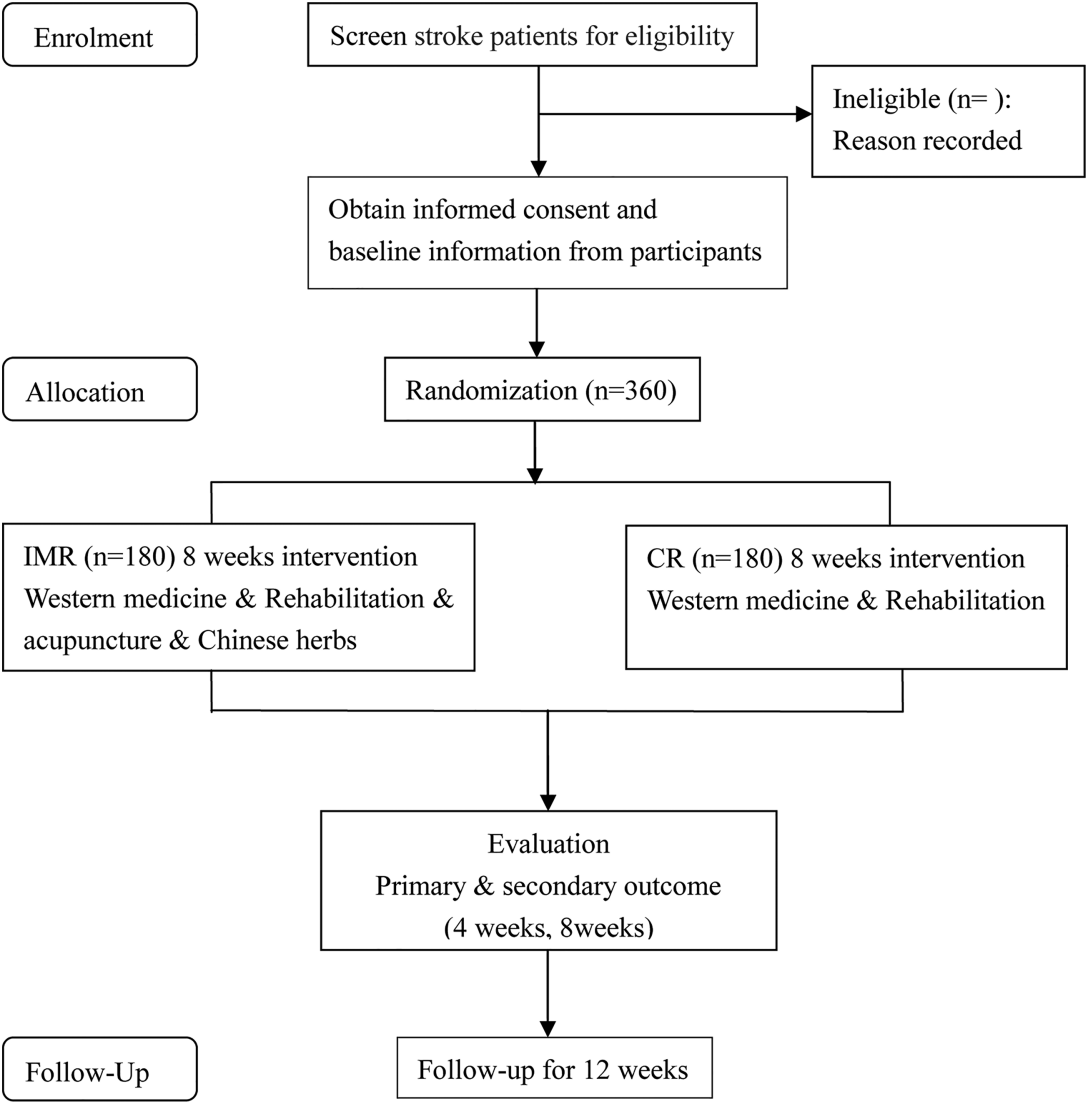
Route diagram of study design. IMR, integrative medicine rehabilitation; CR, conventional rehabilitation.

The study is a clinical research design on integrated rehabilitation with traditional Chinese and Western medicine on subacute stage of stroke in a multicentre, randomised, controlled, assessor-blinded clinical trial. Participants recruited from three large Chinese medical hospitals will be randomly divided into two groups (an IMR group and a CR group) using an Excel generated random numbers list. The CR group will receive basic Western medical treatment and rehabilitation, which includes physical therapy treatment, and/or cognitive training for cognitive impairment, and/or psychological counselling for emotional disorders, 6 days per week. The IMR group will be additionally given acupuncture (30 min of acupuncture therapy daily for 6 days per week lasting 8 weeks) and Chinese herbs (treatment based on syndrome differentiation once a day lasting 8 weeks). The specific route diagram is presented in figure 1.

### Participant recruitment

After getting the approval of the Institutional Review Board, we recruited participants by advertising in local newspapers, health-related TV programmes, Internet, and posters in hospitals and communities. The recruiting time was from 1 March 2012 to 31 December 2014. The patients intending to join the study can consult with study coordinators regarding any questions they may have. Once the patients qualified and agreed to participate in the study, informed consent was obtained prior to running the series of baseline measurement assessments.

### Inclusion criteria

To be eligible, participants must meet the following conditions: (1) patients must be 35–80 years old, with a recent (30–40 days) ischaemic stroke; (2) patients should have a National Institutes of Health Stroke Scale (NIHSS) score between 4 and 24; (3) the stroke should be the first incidence or patients can have a history of stroke, but must be without disability (modified Rankin Scale, mRS score ≤1).

### Exclusion criteria

Participants who conform to any of the following conditions will be excluded: (1) patients received thrombolytic therapy or participated in other clinical trials in the past 3 months; (2) patients suffered from serious heart, liver or kidney-related diseases, blood coagulation dysfunction or severe mental disorders; (3) patients cannot accept acupuncture, and (or) Chinese medicine treatment; (4) patients are pregnant or breast-feeding; (5) patients have congenital disabilities.

### Ethical considerations

Each of the ethics committees of the Third Affiliated Hospital of Zhejiang Chinese Medical University, Hangzhou Hospital of Traditional Chinese Medicine and Jiaxing Hospital of Traditional Chinese Medicine, all approved the study. The purpose, nature and potential risks of the experiments were fully explained to the patients and their families. All patients gave their written and oral informed consent before participating in the study.

### Randomisation and blinding

Randomisation was performed on Excel computer software for the study. The generated list of random numbers was printed, cut into small pieces, separated and placed into sequentially numbered, opaque, sealed envelopes. The envelopes were saved by special screeners. When a participant was included, the screeners opened the envelope to get the group information. Then the subject was informed whether they would be in the treatment group or control group, with or without acupuncture and Chinese herbs. All of the rehabilitation therapists, outcome assessors and data analysts are blinded to group assignments. However, it is impossible to make acupuncturists blinded, because they are trained to perform the treatment for participants of the IMR group.

### Interventions and comparison

The study is a randomised clinical trial carried out in three centres. Participants will be randomised to either the IMR group or CR group. Both groups will receive conventional stroke rehabilitation care, which includes normal limb posture, physiotherapy (PT) and occupational therapy (OT), and/or cognitive training for cognitive impairment, and/or psychological counselling for an emotional disorder. The rehabilitation team develops the rehabilitation programme according to the investigator's brochure. Rehabilitation includes PT and OT for 2 h per day, 6 days per week for each participant. The IMR group will receive 30 additional minutes of acupuncture therapy every day, 6 days per week and take Chinese herbal decoction (twice a day) for 8 weeks during the inpatient stay.

### Acupuncture treatment

The acupuncture programme, developed by experts of our project group after many discussions, was performed by certified acupuncturists with more than 5 years of clinical experience. To ensure the same condition, all of the treatment protocols and processes are detailed below:

Scalp acupuncture: Select filiform needles (size 0.25 mm×40 mm, Huatuo brand, manufactured by Suzhou Medical Appliance in Suzhou, Jiangsu Province, China), swiftly insert the needles subcutaneously at 30° to the scalp on the top midline, the motor area and the sensory area of the affected side.

Body acupuncture (the affected side): LI15 (JianYu), LI11 (QuChi), LI10 (ShouSanLi), SJ5 (WaiGuan), LI4 (HeGu) for upper limbs; ST32 (BiGuan), ST36 (ZuSanLi), GB34 (YangLingQuan), GB39 (XuanZhong), BL60 (KunLun) for lower limbs. Acupoints of the above are referred to the People's Republic of China, State Standard Name and Location of Acupoints (GB 12346-2006).

Modification according to dysfunction after stroke: For cognitive impairment patients, add GV20 (BaiHui), GV24 (ShenTing), GB13 (BenShen), EX-HN1 (SiShenCong), Temple-Three-Needles (which is located in the temple area, on the opposite side of the hemiplaegia, the first needle is located in 2 cun straight above ear apex, then the second and third needles are separately located at the lateral 1 cun of the first needle). For emotional disorder patients, add LR3 (TaiChong), PC6 (NeiGuan), GV20 (BaiHui), GV29 (YinTang), GV24 (ShenTing).

Modification according to syndrome differentiation: For disturbance of wind-fire type, add LR2 (XingJian), LR3 (TaiChong), LR14 (QiMen); For phlegm-stasis blocking collaterals, add SP10 (XueHai), ST40 (FengLong); For yin deficiency and wind act, add SP6 (SanYinJiao), KI3 (TaiXi), LR3 (TaiChong); For qi deficiency and blood stasis type, add CV6 (QiHai), CV4 (GuanYuan), BL17 (GeShu).

Electroacupuncture will be used when the patients De Qi (have the sensation of aching, numbness, tingling or warmth). Then, LI15 (JianYu) and LI11 (QuChi), ST36 (ZuSanLi) and GB39 (XuanZhong) will be connected to GB6805–2 Electro-Acu Stimulators (Huayi Medical Supply & Equipment Co, Ltd, Shanghai, China). The parameter is 2 Hz intermittent wave at the intensity within patients’ tolerance.

### Syndrome differentiation and Chinese herbal medicine

The prescription of Chinese herbs is based on syndrome differentiation. We formulated the treatment protocol through textbooks and ancient literature, as well as experts’ experiences, and the final version of the protocol was used for patients with stroke of three centres before the trial was carried out. There are four types according to syndrome differentiation: (1) For the syndrome of disturbance of wind-fire, the prescription is: Tian Ma Gou Teng decoction modified (Tian Ma 9 g, Gou Teng 15 g, Shi Jue Ming 15 g, Shan Zhi Zi 9 g, Huang Qin 9 g, Chuan Niu Xi 15 g, Du Zhong 12 g, Yi Mu Cao 15 g, Sang Ji Sheng 15 g,Ye Jiao Teng 9 g, Fu Sheng 9 g, raw Long Gu 30 g, raw Mu Li 30 g); (2) For the syndrome of phlegm-stasis blocking collaterals, the prescription is: Ban Xia Bai Zhu Tian Ma decoction and Tao Hong Si Wu decoction modified (Ban Xia 9 g, Bai Zhu 9 g, Tian Ma 9 g, Fu Lin 9 g, Ju Hong 6 g, Sheng Di 15 g, Dang Gui 15 g, Chuan Xiong 9 g, Tao Ren 9 g, Hong Hua 6 g); (3) for the syndrome of yin deficiency and wind act, the prescription is: Zhen Gan Xi Feng decoction modified (raw Long Gu 15 g, raw Mu Li 15 g, Dai Zhe Shi 30 g, Gui Ban 15 g, Bai Shao 15 g, Xuan Shen 15 g, Tian Dong 15 g, Chuan Lian Zi 6 g, Yin Chen 6 g, Chuan Xiong 15 g, raw Mai Ya 6 g, fried Gan Cao 6 g); and (4) for the syndrome of qi deficiency and blood stasis, the prescription is: Bu Yang Huan Wu decoction modified (raw Huang Qi 30 g, Dang Gui 15 g, Tao Ren 6 g, Hong Hua 6 g, Di Long 12 g, Chi Shao 15 g).

### Conventional rehabilitation group

Patients in the CR group do not receive acupuncture and Chinese herbs. This group only receives basic Western medical and rehabilitation treatment, in the same frequency, with the same course of treatment as the IMR group.

### Outcome assessment

The assessment data will be collected at baseline, 4 and 8 weeks postrandomisation, and then 12 weeks after completing the treatment.

### Baseline assessment

Demographic data includes gender, age, nationality, education level, occupation and marital status. Information on stroke risk factors regarding smoking, drinking, height, weight, blood pressure, family history of stroke, blood lipids and blood sugar is gathered through review of the medical records. Several classifications of disease data regarding: rehabilitation evaluation scales on neurological deficit, sensory motor, and cognitive and emotional disorders will be also analysed before randomisation.

### Primary outcome measurement

The primary outcome measure is the Modified Barthel Index (MBI), which was developed in 1955 as a simple index of independence useful in scoring disability.^8^ The MBI scale is a reliable measure of functional independence. It is sensitive and valid to evaluate dependence in the activities of daily living (ADL). It includes 10 variables (defecating, urinating, feeding, bathing, grooming, dressing, toileting, transfer, walking and using stairs) describing ADL and mobility. A higher number is associated with a greater likelihood of being able to live at home with a degree of independence. This index is used as a standardised assessment on rehabilitation wards and also as standardised follow-up assessment to determine whether gains achieved by patients with stroke while hospitalised are maintained after discharge.^9–11^

### Secondary outcome measures

#### The NIHSS for neurological deficits

The NIHSS is a graded neurological examination that assesses consciousness, best gaze, visual field, facial palsy, motor arm, motor leg, limb ataxia, sensory, best language, dysphagia and neglect. The scale was developed for use in acute-stroke trials, and has since been widely used as a standard part of the assessment in clinical trials. Its scores range from 0 to 42, with scores above 25 indicating very severe neurological impairment, scores of 5–24 suggesting moderately severe to severe impairment, and scores below 5 indicate mild impairment.^12^ ^13^

#### The Fugl-Meyer Assessment scale for motor dysfunction

The Fugl-Meyer Assessment (FMA) was developed as the first quantitative evaluative instrument for measuring sensorimotor stroke recovery, which includes items dealing with the shoulder, elbow, forearm, wrist and hand in the upper extremity (UE, 66 points), and the hip, knee and ankle in the lower extremity (34 points).^14^ The motor domain has well-established reliability and validity as an indicator of motor impairment severity across different stroke recovery time points.^15^

#### The mini-mental state examination and Montreal Cognitive Assessment (MoCA) for cognitive impairment

Cognitive function is assessed by the mini-mental state examination (MMSE) and MoCA scale. MMSE is a brief wide-range screening test with 30 aggregate scores, which is more suitable for uneducated or old populations. It assesses memory, orientation, calculation attention span, and ability to express and to read. In fact, MMSE is widely used because of its high specificity, but it cannot subtly detect patients with mild cognitive impairment (MCI), whose scores are in the normal range.^16^ In contrast, MoCA is more sensitive. It is also a test with 30 points, of which items include visual-spatial abilities, executive functions, attention span, concentration, memory, language and orientation. The MoCA detects patients with MCI with 90% sensitivity and 87% specificity.^17^

#### The Self-Rating Depression Scale and Hamilton's Depression Scale for emotional disorder

Self-Rating Depression Scale (SDS) is a self-report instrument covering 20 items, either positive or negative, with a four-point scale ranging from 1 to 4. The standardised score is the total score times 1.25, which results in 25–100.^18^ Furthermore, in antidepressant clinical trials, the Hamilton Depression Rating Scale has been the ‘gold standard’ for use.^19^ The Hamilton's Depression Scale (HAMD) Scale we selected includes 24 items, consisting of sense of guilt, sleeping problems, suicide, lack of interest, anxiety, loss of weight, self-abasement, hopelessness, and so on; 14 items with a score of 0–4 and 10 items with a score of 0–2. The range of scores for the whole test is 0–76. Higher scores indicate a higher level of mental disorder.^20^

### The incidence of adverse events

Participants are to be questioned and report all adverse events (AEs) at each visit point, and all AEs reports will be recorded and assessed by the investigators. If serious AEs occur, the researchers should report to the principal investigator and ethics committee immediately, who will make a decision on whether or not the participant needs to withdraw from the study. If the participant suffered serious AEs, unbinding is permissible and procedure is followed for revealing a participant's allocated intervention during the trial. Compensation will be provided to those who suffer harm from trial participation.

In order to assess the safety of herbal medicine, we will perform the following tests on participants of the IMR group at baseline (week 0) and after treatment (week 12): routine blood test, routine urine test, routine faeces test, kidney function test and liver function test. In addition, investigators will ask subjects at each visit whether they have experienced allergies or gastrointestinal discomfort during the study period.

The AEs of acupuncture may include local bleeding, haematoma, pallor, sweating or dizziness, fainting during the acupuncture treatment, unbearable prickling or retained needle after treatment. The investigator should record the date of occurrence, time, degree, measurement related to the treatment and consequence.

### Quality control and data management

This is a 20-week clinical trial, in which participants need to take herbal medicine and acupuncture for 8 weeks, and accept a 12-week follow-up, attend four assessment visits (rehabilitation evaluation), obtain one set of laboratory tests (safety assessments). Before the study, the trial protocol has been reviewed and revised by experts on acupuncture, neurology, rehabilitation, statistics and methodology several times. All the members belonging to the trial are asked to take part in a series of training to ensure that the personnel involved fully understand the research protocol and standard operating procedures for the study. During the study, the Clinical Research Institute of Zhejiang Provincial is responsible for generating the allocation sequence, quality control, and censors make regular visits (once a month) to monitor for protocol violations, the recruitment rate, AEs and participant compliance. This clinical trial is independent from sponsors and competing interests. The clinical coordinators of three centres are specifically designated to enrol participants and assign participants to interventions, but not to participate in treatment and assessment for participants. Outcome assessors and data analysts will be blinded after assignment to interventions, without access to patients’ group information.

The measurements are mainly rehabilitation evaluations. All of the data will first be recorded on the paper version of the case report form by assessors, then double entered into the EDC system electronically. A specified statistics centre of the Clinical Research Institute of Zhejiang Province will be responsible for data management. All data will be double entered to ensure accuracy. The source of any inconsistencies will be explored and resolved.

### Sample size calculation

The primary efficacy parameter is the change in MBI scores from baseline to the end of treatment after 8 weeks. Sample size calculations are based on our preliminary test and previous study.^21^ ^22^ The expected difference between CR group and IMR group is a 10 value, that is to say the score of MBI of the IMR group was a 10 value higher than that of the CR group, and the SD is about 31. A two sided 5% significance level and 80% power were considered, and the following equation was used:
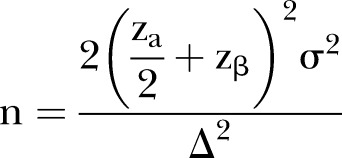


Approximately 150 participants in each group were calculated to be required. Estimating a 20% dropout rate, each group will require 180 initial participants.

### Statistical analysis

Efficacy and safety analyses will be conducted according to the intention-to-treat (ITT) principle by a statistician blinded to group allocation. Missing values will be imputed by the last-observation-carried-forward method. All statistical analyses will be performed using Statistical Product and Service Solutions (SPSS) statistical package program (V.17.0, SPSS Inc, Chicago, Illinois, USA). The primary outcomes (MBI) and FMA will undergo ITT analysis including all the patients who are randomised. The analysis of cognitive impairment and emotional disorder will be made among the defined population of corresponding dysfunction. Continuous variables will be expressed as means with SDs. For normally distributed variables, two independent samples will be compared by independent sample t test. On the other hand, for abnormally distributed variables, non-parametric tests will be used and the data will be expressed as medians with ranges. A p value of less than 0.05 is considered as statistical significance. Safety analysis is based on the frequency of AEs relating to the treatment.

## Discussion

China’s extensive clinical experience in the use of TCMs in stroke therapy indicates that TCM preparations are effective. More than 100 traditional medicines are currently in use for stroke therapy in China.^23^ However, insufficient good-quality evidence on the effects of TCM in ischaemic stroke exists on the primary outcome.^24^ One possibility for lack of evidence in the literature could result from the significant clinical and methodological heterogeneity, preventing effective meta-analysis techniques. No meta-analysis has been performed and thus no cumulative results obtained by pooling RCT data exist.^25^ Further randomised controlled trials are justified.

Acupuncture is recommended for stroke according to the WHO.^26^ Literature reviews have demonstrated the safety of acupuncture, legitimising its ethical use for patients, without causing harm.^7^ Despite its safety, the limited availability of rigorous RCTs and the lack of research available on complementary and alternative medicine treatments such as acupuncture, create a controversial opinion on its benefit for specific disease outcomes.^7^ ^27^ ^28^ This lack of evidence necessitates additional RCT study on the clinical efficacy of IM on stroke outcomes.

Complementary or alternative medicine such as acupuncture and Chinese medicine, or IM, has become increasingly prevalent and popular, not only in China, but also worldwide.^29^ ^30^ Integrated traditional Chinese and Western medicine for stroke rehabilitation is widely used in China, making it an ideal setting to study stroke treatment protocols. The integrated approach is forming characteristics of some of China's stroke treatment modalities, which can be observed as a model for the rest of the world.^31^ In China, many patients with stroke receive basic Western medicine and rehabilitation as well as acupuncture and Chinese medicine during hospital stays. So we are conducting this clinical trial, which is close to the actual treatment strategy in China, to objectively evaluate the clinical efficacy. Syndrome differentiation and treatment is the essence of Chinese medicine, so in our study, Chinese herbal prescriptions for patients with stroke are clarified. Four types of herbal medicine are most common in the clinic according to syndrome differentiation. The acupuncture programme traditionally includes scalp acupuncture and body acupuncture and, in addition, some acupoints are selected according to the patient’s dysfunction and also syndrome differentiation. Under strict quality control, this study could potentially confirm whether or not IM is an effective adjunct to the standard rehabilitation therapy for ischaemic stroke. Our study may also assess the efficacy of IM in promoting the recovery of motor dysfunction, cognitive impairment and emotional disorder.

## Conclusion

This trial has been designed to provide robust data on the efficacy of IM for patients with ischaemic stroke. It is also expected to clarify whether or not IM is effective for motor, cognitive or emotional disorder after stroke.

## Supplementary Material

Author's manuscript

Reviewer comments
