# Supplementary material for: Integrative medicine for subacute stroke rehabilitation: a study protocol for a multicentre, randomised, controlled trial
Source: BMJ Open. 2014 Dec 4;4(12):e007080. doi: 10.1136/bmjopen-2014-007080 (PMC4256536; doi:10.1136/bmjopen-2014-007080)
Supplement: Author's manuscript [file bmjopen-2014-007080.draft_revisions.pdf]

Integrative Medicine for Sub-acute Stroke Rehabilitation: Study Protocol for a Multicenter, Randomized, Controlled Trial

Journal:	*BMJ Open*
Manuscript ID:	bmjopen-2014-007080
Article Type:	Protocol
Date Submitted by the Author:	04-Nov-2014
Complete List of Authors:	Fang, Jianqiao; The Third Affiliated Hospital of Zhejiang Chinese Medical University, Department of Acupuncture; Zhejiang Chinese Medical University, The Third Clinical Medical College<br>Chen, Lifang; The Third Affiliated Hospital of Zhejiang Chinese Medical University, Department of Acupuncture<br>Chen, Luni; Zhejiang Chinese Medical University, The Third Clinical Medical College<br>Wang, Chao; Zhejiang Chinese Medical University, The Third Clinical Medical College<br>Keeler, Crystal; Five Branches University, Innovations to Wellness<br>Ma, Ruijie; The Third Affiliated Hospital of Zhejiang Cheinses Medical University, Department of Acupuncture<br>Xu, Shouyu; The Third Affiliated Hospital of Zhejiang Chinese Medical University, Department of Rehabilitation<br>Shen, Laihua; Jiaxing Hospital of Traditional Chinese Medicine, Department of Acupuncture & Encephalopathy<br>Bao, Yehua; Hangzhou Hospital of Traditional Chinese Medicine, Department of Acupuncture & Rehabilitation<br>Ji, Conghua; Zhejiang Provincial Hospital of Traditional Chinese Medicine, The Clinical Research Institute
**Primary Subject Heading**:	Complementary medicine
Secondary Subject Heading:	Complementary medicine
Keywords:	STROKE MEDICINE, COMPLEMENTARY MEDICINE, Rehabilitation medicine < INTERNAL MEDICINE

SCHOLARONE™ Manuscripts

Integrative Medicine for Sub-acute Stroke Rehabilitation: Study Protocol for a Multicenter, Randomized, Controlled Trial

Jianqiao Fang,[1,2*] Lifang Chen,[1] Luni Chen,[2] Chao Wang,[2] Crystal Lynn Keeler,[3] Ruijie Ma,[1] Shouyu Xu,[4] Laihua Shen,[5] Yehua Bao,[6] Conghua Ji,[7]

Correspondence to
Professor Jianqiao Fang[1,2*];
fangjianqiao7532@163.com

Lifang Chen[1]
Email: clfang@163.com

Luni Chen[2]
Email: clnhz@126.com

Chao Wang[2]
wangchaozy1126@163.com

Crystal Lynn Keeler[3]
Email: Crystal.clk@gmail.com

Ruijie Ma[1]
Email: maria7878@sina.com

Shouyu Xu[4]
Email: overnightjo@msn.com

Laihua Shen[5]
Email: slh86ly@163.com

Yehua Bao[6]
Email: byh@hz.cn

Conghua Ji[7]
Email: jchi_2005@163.com

For peer review only

ABSTRACT

Introduction: Many stroke patients receive integrative medicine in China, which includes the basic treatment of Western medicine and routine rehabilitation, in conjunction with acupuncture and Chinese medicine. The question whether integrative medicine is efficacious for stroke rehabilitation is still controversial and very little research currently exists on the integrated approach for this condition. Consequently, we will conduct a multicenter, randomized, controlled, assessor-blinded clinical trial to assess the effectiveness of integrative medicine on stroke rehabilitation.

Methods and analysis: 360 participants recruited from three large Chinese medical hospitals in Zhejiang Province will be randomly divided into the integrative medicine rehabilitation (IMR) group and the conventional rehabilitation (CR) group in a 1:1 ratio. Participants in IMR group will receive acupuncture and Chinese herbs in addition to basic western medicine and rehabilitation treatment. The CR group will not receive acupuncture and Chinese herbal medicine. The assessment data will be collected at baseline, 4 and 8 weeks post-randomization, and then at 12-weeks follow-up. The primary outcome assessment tool, the Modified Barthel Index (MBI), will be used to evaluate activities of daily living (ADL). The secondary outcomes will be measured by the National Institutes of Health Stroke Scale (NIHSS) for neurologic deficits, Fugl-Meyer Assessment (FMA) for motor dysfunction, and the Mini-Mental State Examination（MMSE）and Montreal Cognitive Assessment（MoCA）for cognitive impairment. For emotional disorders, the Hamilton's Depression Scale (HAMD) and Self-Rating Depression Scale (SDS) will measure emotional state, and the incidence of adverse events (AEs) will also be observed.

Ethics and dissemination: Ethical approval was obtained from ethics committees of the Third Affiliated Hospital of Zhejiang Chinese Medical University , the Hangzhou Hospital of Traditional Chinese Medicine and the Jiaxing Hospital of Traditional Chinese Medicine. The results will be disseminated in a peer-reviewed journal and presented at international congresses.

For peer review only

Trial registration: Chinese Clinical Trial Register: ChiCTR-TRC-12001972, http://www.chictr.org/usercenter/project/edit.aspx?proj=2561

Keywords Integrative Medicine, Sub-acute Stroke, Study Protocol, Randomized Controlled Trial

For peer review only

INTRODUCTION

Stroke is the second most common cause of death and leading cause of adult disability worldwide[1], The number of patients who die from stroke is more than three times that from coronary heart disease[2]. Modern Western medicine in China undoubtedly occupies the dominant position in prevention and treatment of stroke. However, most patients with stroke are treated with one or more types of Traditional Chinese Medicine (TCM) in addition to Western medicine. The role of TCM should not be ignored[3-5]. With the development of integrative medicine, which was established in the 1980s, more and more stroke patients receive integrative medicine treatment nowadays. The main integrative treatment includes the standard Western medicine and rehabilitation for stroke, as well as acupuncture and (or) Chinese herbs[6]. Nevertheless, there is not yet enough evidence to show the effect of integrative medicine for stroke. More rigorously designed, large scale, multicenter randomized trials are necessary, to assess the effectiveness of integrative medicine on stroke rehabilitation[7].

METHODS

Study design

The study is a clinical research design on integrated rehabilitation with traditional Chinese and Western medicine on sub-acute stage of stroke, in a multicenter, randomized, controlled, assessor-blinded clinical trial. Participants recruited from three large Chinese medical hospitals, will be randomly divided into two groups (IMR group and CR group) using Statistical Product and Service Solutions (SPSS) statistical package program (ver. 17.0, SPSS Inc., Chicago, IL, USA) software. The CR group will receive basic Western medical treatment and rehabilitation, which includes physical treatment, and/or cognitive training for cognitive impairment, and/or psychological counseling for emotional disorders, six days per week. The IMR group will be additionally given acupuncture (thirty minutes of acupuncture therapy daily for six days per week lasting eight weeks) and Chinese herbs (treatment based

For peer review only

on syndrome differentiation for once a day lasting eight weeks). The specific route diagram is presented in figure 1.

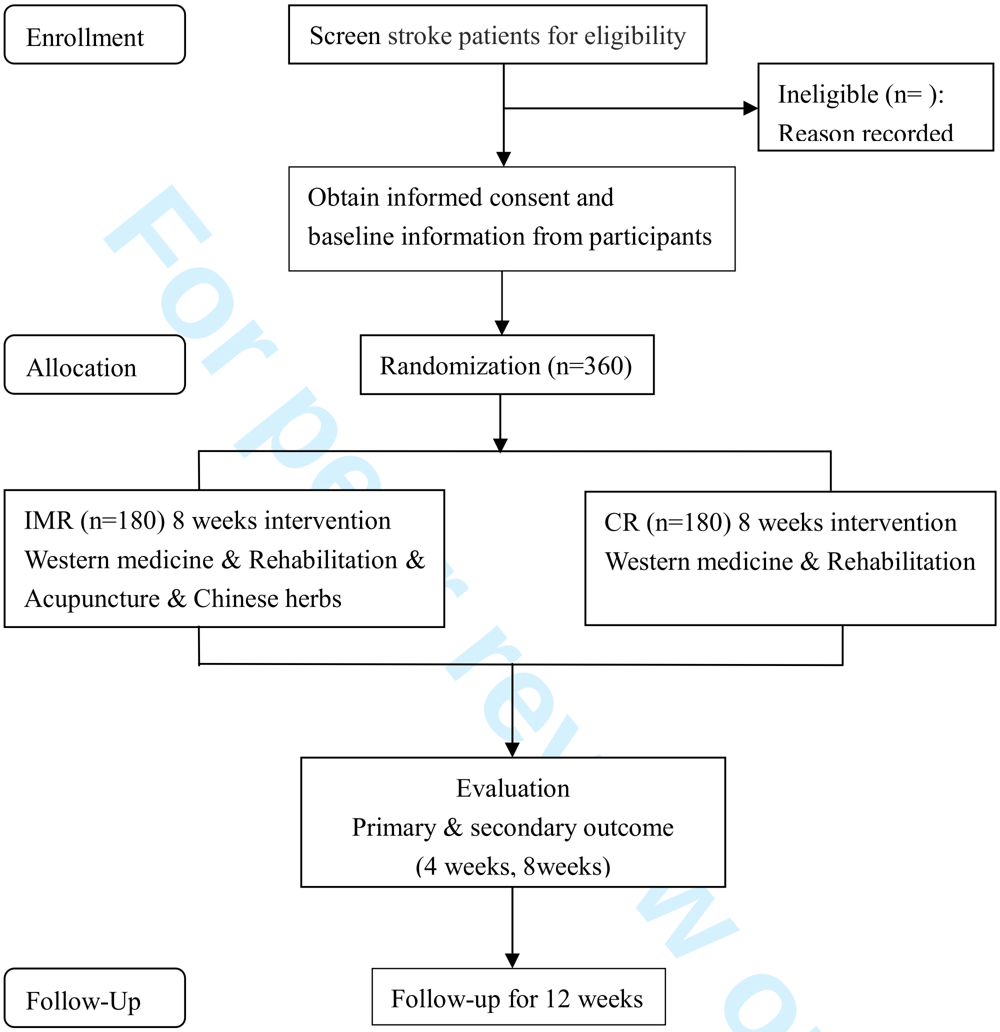

Figure 1.Route diagram of study design.

Participant recruitment

After getting the approval of Institutional Review Board, we recruited participants by advertising in local newspapers, health-related TV program, Internet and posters in hospitals and communities. The recruiting time was from March 1, 2012 to December 31, 2014. The patients intending to join the study can consult with study coordinators regarding any questions they may have. Once the patients qualified and agreed to participate in the study, informed consent was obtained prior to running the series of baseline measurement assessments.

For peer review only

Inclusion criteria

To be eligible, participants must meet the following conditions: (1) patients of 35-80 years old, with a recent (30～40 days) ischemic stroke; (2) patients with a NIHSS score of between 4 and 24; (3) The stroke should be the first incidence or patients can have a history of stroke, but without disability (modified Rankin Scale, mRS score ≤1).

Exclusion criteria

Participants who conform to any conditions below will be excluded: (1) Patients received thrombolytic therapy and participated in other clinical trials in last three months; (2) Patients who suffered from serious heart, liver, kidney related diseases, blood coagulation dysfunction, or severe mental disorders; (3) Patients who can not accept acupuncture, and(or) Chinese medicine treatment; (4) Women who are pregnant or breast-feeding.

Ethical considerations

Each of the ethics committees of the Third Affiliated Hospital of Zhejiang Chinese Medical University , Hangzhou Hospital of Traditional Chinese Medicine and Jiaxing Hospital of Traditional Chinese Medicine, all approved the study. The purpose, nature, and potential risks of the experiments were fully explained to the patients and their families. All patients gave their written and oral informed consent before participating in the study.

Randomization and blinding

Randomization was done by computer software in the study. The generated random numbers was recorded in a small paper, which has been saved in sequentially numbered, opaque, sealed envelope. The envelopes were saved by the special screeners. When the participant is included, the screeners will open the envelope to get the group information. Then the subject was informed whether they will be in the treatment group or control group, with or without acupuncture & Chinese herbs. All of the rehabilitation therapists, outcome assessors, and data analysts are blinded to group assignments. However, it is impossible to make acupuncturists blinded, because they are trained to perform the treatment for participants of IMR group.

For peer review only

Interventions and comparison

The study is a randomized clinical trial carried out in three arms. Participants will be randomized to either the IMR group or CR group. Both groups will receive conventional stroke rehabilitation care, which includes normal limb posture, physiotherapy (PT) and occupational therapy (OT), and/or cognitive training for cognitive impairment, and/or psychological counseling for emotional disorder. The rehabilitation team develops the rehabilitation program according to the investigator's brochure. Rehabilitation includes physiotherapy and occupational therapy for two hours per day, six days per week for each participant. The IMR group will receive 30 additional minutes of acupuncture therapy every day, six days per week and take Chinese herbal decoction (twice a day) for eight weeks during the inpatient stay.

Acupuncture treatment

Acupuncture program is developed by experts of our project group, which has been discussed many times and performed by certified acupuncturists with more than five years of clinical experience. To ensure the same condition, all of the treatment protocols and processes will be written in detail as follows:

Scalp acupuncture: Select filiform needle (size 0.25 mm × 40 mm, Huatuo brand, manufactured by Suzhou Medical Appliance in Suzhou, Jiangsu Province China), swiftly insert the needle subcutaneously in 30° to the scalp on the top midline, the motor area and the sensory area of the affected side.

Body acupuncture (the affected side): LI15 (JianYu), LI11 (QuChi), LI10 (ShouSanLi), SJ5 (WaiGuan), LI4 (HeGu) for upper limbs; ST32 (BiGuan), St36 (ZuSanLi), GB34 (YangLingQuan), GB39 (XuanZhong), BL60 (KunLun) for lower limbs. Acupoints of the above are referred to the People's Republic of China, State Standard Name and Location of Acupoints (GB 12346-2006).

Modification according to dysfunction after stroke: For cognitive impairment patients, add GV20 (BaiHui), GV24 (ShenTing), GB13(BenShen), EX-HN1 (SiShenCong), Temple-Three-Needles (which is located in temple area, on the opposite side of hemiplegia, the first needle is located in 2 cun straight above ear apex, then the second and third needles are separately at the lateral 1 cun of the first needle).

For peer review only

For emotional disorder patients, add LR3 (TaiChong), PC6 (NeiGuan), GV20 (BaiHui), GV29 (Yin Tang), GV24 (Shen Ting).

Modification according to syndrome differentiation: For disturbance of wind-fire type, add LR2 (Xing Jian), LR3 (Tai Chong), LR14(Qi Men); For phlegm-stasis blocking collaterals, add SP10(Xue Hai), ST40(Feng Long); For yin deficiency and wind act, add SP6 (San Yin Jiao), KI3(Tai Xi), LR3 (Tai Chong); For qi deficiency and blood stasis type, add CV6(Qi Hai), CV4(Guan Yuan), BL17(Ge Shu).

Electro-acupuncture will be used when the patients De Qi (the sensation of aching, numbness, tingling or warmth). Then, LI15 (Jian Yu) and LI11 (Qu Chi), St36 (Zu San Li) and GB39 (XuanZhong) will be connected to GB6805-2 Electro-Acu Stimulators (Huayi Medical Supply & Equipment Co., Ltd, Shanghai, China). The parameter is 2Hz intermittent wave at the intensity within patients' tolerance.

Syndrome differentiation and Chinese herbal medicine

The prescription of Chinese herbs is based on syndrome differentiation, we formulated the treatment protocol through a systematic review, textbooks and ancient literatures, as well as experts' experiences, and the final version of the protocol was used for stroke patients of three centers before the trial carried out. There are four types according to syndrome differentiation: (1)For the syndrome of disturbance of wind-fire, Prescription: Tian Ma Gou Teng decoction modified (Tian Ma 9g, Gou Teng 15g, Shi Jue Ming 15g, Shan Zhi Zi 9g, Huang Qin 9g, Chuan Niu Xi 15g, Du Zhong 12g, Yi Mu Cao 15g, Sang Ji Sheng 15g,Ye Jiao Teng 9g, Fu Sheng 9g, raw Long Gu 30g, raw Mu Li 30g); (2)For the syndrome of phlegm-stasis blocking collaterals, Prescription: Ban Xia Bai Zhu Tian Ma decoction and Tao Hong Si Wu decoction modified (Ban Xia 9g, Bai Zhu 9g, Tian Ma 9g, Fu Lin 9g, Ju Hong 6g, Sheng Di 15g, Dang Gui 15g, Chuan Xiong 9g, Tao Ren 9g, Hong Hua 6g) (3)For the syndrome of yin deficiency and wind act, Prescription: Zhen Gan Xi Feng decoction modified (raw Long Gu 15g, raw Mu Li 15g, Dai Zhe Shi 30g, Gui Ban 15g, Bai Shao 15g, Xuan Shen 15g, Tian Dong 15g, Chuan Lian Zi 6g, Yin Chen 6g, Chuan Xiong 15g, raw Mai Ya 6g, fried Gan Cao 6g) (4)To syndrome of qi deficiency and blood stasis, Prescription: Bu Yang Huan Wu decoction modified (raw Huang Qi 30g,

For peer review only

Dang Gui 15g, Tao Ren 6g, Hong Hua 6g, Di Long 12g, Chi Shao 15g).

Conventional Rehabilitation Group

Patients in CR group do not receive acupuncture and Chinese herbs. This group only receives basic Western medical and rehabilitation treatment, in the same frequency, with the same course of treatment as the IMR group.

Outcome assessment

The assessment data will be collected at baseline, 4 and 8 weeks post-randomization, and then 12 weeks after completing the treatment.

Baseline assessment

Demographic data includes gender, age, nationality, education level, occupation, and marital status. Information on stroke risk factors regarding smoking, drinking, height, weight, blood pressure, family history of stroke, blood lipids, and blood sugar is gathered through review of the medical records. Several classifications of disease data regarding: rehabilitation evaluation scales on neurological deficit, sensory motor, cognitive, and emotional disorders will be also analyzed before randomization.

Primary outcome measurement

The primary outcome measurement is the Modified Barthel Index (MBI), which was developed in 1955 as a simple index of independence useful in scoring disability[8]. The MBI scale is a reliable measure of functional independence. It is sensitive and valid to evaluate dependence in the activities of daily living (ADL). It includes ten variables (defecating, urinating, feeding, bathing, grooming, dressing, toileting, transfer, walking, and stairs) describing ADL and mobility. A higher number is associated with a greater likelihood of being able to live at home with a degree of independence. This index is used as a standardized assessment on rehabilitation wards and also as standardized follow-up assessment to determine whether gains achieved by patients with stroke while hospitalized are maintained after discharge[9-11].

Secondary outcome measures

The National Institutes of Health Stroke Scale (NIHSS) for neurologic deficits

The National Institutes of Health Stroke Scale (NIHSS) is a graded neurological examination that assesses consciousness, best gaze, visual field, facial palsy, motor

For peer review only

arm, motor leg, limb ataxia, sensory, best language, dysphagia, and neglect. The scale was developed for use in acute-stroke trials, and has since been widely used as a standard part of the assessment in clinical trials. Its scores range is from 0 to 42, with scores above 25 indicating very severe neurological impairment, scores of 5 to 24 suggesting moderately severe to severe impairment, and scores below 5 indicate mild impairment[12-13].

The Fugl-Meyer Assessment (FMA) scale for motor dysfunction

The FMA was developed as the first quantitative evaluative instrument for measuring sensorimotor stroke recovery, which includes items dealing with the shoulder, elbow, forearm, wrist, and hand in the upper extremity (UE, 66 points) and the hip, knee, and ankle in the lower extremity(LE, 34 points)[14].The motor domain has well-established reliability and validity as an indicator of motor impairment severity across different stroke recovery time points[15].

The Mini-Mental State Examination（MMSE）and Montreal Cognitive Assessment（MoCA）for cognitive impairment

Cognitive function is assessed by MMSE and MoCA scale. MMSE is a brief wide-range screening test with 30 aggregate scores, which is more suitable for uneducated or old populations. It assesses memory, orientation, calculation, attention, expressing, and reading. In fact, MMSE is widely used because of its high specificity, but it can't subtly detect mild cognitive impairment (MCI) patients, whose scores are in the normal range[16]. In contrast, MoCA is more sensitive. It is also a test with 30 points, of which items including visual-spatial abilities, executive functions, attention, concentration, memory, language, orientation. The MoCA detects MCI patients with 90% sensitivity and 87% specificity[17].

The Self-Rating Depression Scale (SDS) and Hamilton's Depression Scale (HAMD) for emotional disorder

SDS is a self-report instrument covering 20 items, either positive or negative, with a four-point scale ranging from 1 to 4. The standardized score is the total score times 1.25, which results in 25 to 100[18]. Furthermore, in antidepressant clinical trials, the Hamilton Depression Rating Scale has been the "gold standard" for use[19]. The

For peer review only

HAMD Scale we selected includes 24 items, consisting of sense of guilt, sleeping problems, suicide, interest, anxiety, loss of weight, self-abasement, hopelessness, and so on, 14 items with a score of 0-4 and 10 items with score of 0-2. The range of scores for the whole test is 0-76. Higher scores indicate a higher level of mental disorder, respectively[20].

The incidence of adverse events

Participants are to be questioned and report all adverse events (AEs) at each visit point, and all AEs reports will be recorded and assessed by the investigators. If serious adverse events happen, the researchers should report to the principal investigator and ethics committee immediately, who will make a decision on whether the participant needs to withdraw from the study.

In order to assess the safety of herbal medicine, we will perform the following tests on participants of IMR group at the baseline (week 0) and after treatment (week 12): blood routine test, urine routine test, feces routine test, kidney function test, liver function test. In addition, investigators will ask every subject at each visit whether they have experienced allergies or gastrointestinal discomfort during the study period.

The adverse events of acupuncture may include local bleeding, hematoma, pallor, sweating or dizziness, fainting during the acupuncture treatment, unbearable prickling, retained needle after treatment. The investigator should record the date of occurrence, time, degree, measurement related to the treatment, and consequence.

Quality control and data management

This is a 20-week clinical trial, in which participants need to take herbal medicine and acupuncture for 8 weeks, and accept a 12-week follow-up, attend four assessment visits (rehabilitation evaluation), obtain one set of laboratory tests (safety assessments), Before the study, the trial protocol has been reviewed and revised by experts on acupuncture, neurology, rehabilitation, statistics, and methodology several times. All the members belonging to the trial are asked to take part in a series of training to ensure that the personnel involved fully understand the research protocol and standard operating procedures for the study. During the study, the Clinical Research Institute of Zhejiang Provincial is responsible for quality control, and

For peer review only

censors have regular visits (once a month) to monitor protocol violations, the recruitment rate, AEs and participant compliance. A specified statistics centre will responsible for data entering and management. All data will be double entered to ensure the accuracy and the source of any inconsistencies will be explored and resolved.

Sample size calculation

The primary efficacy parameter is the change in MBI scores from baseline to the end of treatment after eight weeks. Sample size calculations are based on our preliminary test and previous study[21-22], The expected difference between CR group and IMR group is 10 value, it is to say the score of MBI of IMR group was 10 value higher than that of CR group, and the standard deviation is about 31, A two-sided 5% significance level and 80% power were considered, and the following equation was used:

$$n = \frac{2\left(z_{a}/2 + z_{\beta}\right)^{2}\sigma^{2}}{\Delta^{2}}$$

Approximately 150 participants in each group were calculated to be required. Estimating a 20% dropout rate, each group will require 180 initial participants.

Statistical analysis

Efficacy and safety analyses will be conducted according to the intention-to-treat (ITT) principle by a statistician blinded to group allocation. Missing values will be imputed by the last-observation-carried-forward method. All statistical analyses will be performed using SPSS 17.0. The primary outcomes (MBI) and FMA will undergo ITT analysis including all the patients who are randomized. The analysis of cognitive impairment, emotional disorder will be made among the defined population of corresponding dysfunction. Continuous variables will be expressed as means with standard deviations. For normally distributed variables, two independent samples will be compared by t-test. On the other hand, for abnormally distributed variables, Non-parametric tests will be used, and the data will be expressed as medians with ranges. A P value of less than 0.05 is considered as statistical significance. Safety analysis is based on the frequency of adverse events relating to the treatment.

For peer review only

DISCUSSION

China's extensive experience in the use of traditional Chinese medicines in stroke therapy indicates that TCM preparations are effective, there are more than 100 traditional medicines in use for stroke therapy in China[23]. But there was insufficient good quality evidence on the effects of TCM in ischemic stroke on the primary outcome[24]. Or because of the significant clinical and methodological heterogeneity, no meta-analysis was performed and thus no cumulative result was obtained pooling data of RCTs[25]. Further randomized controlled trials are justified.

Acupuncture is recommended for stroke according to the World Health Organization[26]. However, reviews have shown that although acupuncture appeared to be safe, there is controversy of its benefit[7, 27-28].

Complementary or termed alternative medicine such as acupuncture and Chinese medicine, or integrative medicine has become increasingly prevalent and popular[29-30]. Integrated traditional Chinese and Western medicine for stroke rehabilitation is widely used in China. The integrated approach is forming some China characteristics of stroke treatment mode[31]. Many patients with stroke receive basic Western medicine and rehabilitation as well as acupuncture and Chinese medicine during hospital stays. So we conducted this clinical trial, which is close to the actual treatment strategy in China, to objectively evaluate the clinical efficacy. Syndrome differentiation and treatment is the essence of Chinese medicine, so in our study, Chinese herbal prescriptions for stroke patients are clarified. Four types of herbal medicine are most common in clinic according to syndrome differentiation. The acupuncture program traditionally includes scalp acupuncture and body acupuncture, and in addition some acupoints are selected according to the patient's dysfunction and also syndrome differentiation. Under strict quality control, this study could potentially confirm whether or not integrative medicine (IM) is an effective adjunct to the standard rehabilitation therapy for ischemic stroke. Our study may also assess the efficacy of IM in promoting the recovery of motor dysfunction, cognitive impairment and emotional disorder.

CONCLUSION

For peer review only

This trial has been designed to provide robust data on the efficacy of integrative medicine for patients with ischemic stroke. It is also expected to clarify whether or not integrative medicine is effective for motor, cognitive, or emotional disorder after stroke.

Trial status

The trial is currently enrolling participants; recruitment started in March 1, 2012 and will be completed in December 31, 2014.

List of abbreviations used

FMA, Fugl-Meyer Assessment; MCI, Mild Cognitive Impairment; MMSE, Mini-Mental State Examination; MoCA, Montreal Cognitive Assessment; NIHSS, National Institutes of Health Stroke Scale; VFSS, Video-fluoroscopic Swallowing Study; SPSS, Statistical Product and Service Solutions; mRS, modified Rankin Scale; IMR, integrative medicine rehabilitation; CR, conventional rehabilitation; MBI, Modified Barthel Index; ADL, activities of daily living; HAMD, Hamilton's Depression Scale; SDS, Self-Rating Depression Scale; AEs, adverse events; TCM, Traditional Chinese Medicine; PT, physiotherapy; OT, occupational therapy; IM, integrative medicine.

Competing interests

The authors declare that they have no competing interests.

Authors' contributions

JQF, LFC, LNC, CW, CLK and CHJ and participated in the conception and design of the trial, planning the analysis of the data and drafting the manuscript. RJM, SYX, LHS, and YHB participated in data collection and are in charge of recruitment and treatment of patients in each center. All the authors discussed, revised and approved the final manuscript.

Authors' information

[1] Department of Acupuncture, The Third Affiliated Hospital of Zhejiang Chinese Medical University , No. 219 Moganshan Road, XiHu District, Hangzhou, Zhejiang Province 310005, China

For peer review only

[2] The Third Clinical Medical College of Zhejiang Chinese Medical University , No. 548 Binwen Road, Binjiang District, Hangzhou, Zhejiang Province 310053, China

[3] Innovations to Wellness. Affiliated with Five Branches University. 3031 Tisch Way, 5th floor Administration. San Jose, CA, 95128.

[4] Department of Rehabilitation, The Third Affiliated Hospital of Zhejiang Chinese Medical University , No. 219 Moganshan Road, XiHu District, Hangzhou, Zhejiang Province 310005, China

[5] Department of Acupuncture & Encephalopathy, Jiaxing Hospital of Traditional Chinese Medicine. No. 1501 Zhongshan East Road, Jiaxing, Zhejiang Province 310012, China

[6] Department of Acupuncture & Rehabilitation, Hangzhou Hospital of Traditional Chinese Medicine. No. 453 Tiyuchang Road, XiHu District, Hangzhou, Zhejiang Province 310007, China

[7] The Clinical Research Institute of Zhejiang Provincial Hospital of Traditional Chinese Medicine, No. 54 Youdian Road, Hangzhou, Zhejiang Province 310006, China

Acknowledgements and Funding

This study is funded by a project grant from the Provincial Administration of Traditional Chinese Medicine of Zhejiang (2011ZGG003). The team would like to thank three postgraduate students who contributed their time to the preliminary experiments (Pei Luo, Wei Dong, and Lu Zhang). We also appreciate the help and effort from the people participating in this trial.

For peer review only

References

1. Bonita R, Mendis S, Truelsen T, *et al*. The global stroke initiative. *The Lancet Neurology* 2004; 3(7):391–393.
2. Wang L, Kong L, Wu F, *et al*. Preventing chronic diseases in China. *The Lancet* 2005; 366(9499):1821–1824.
3. Liu M, Wu B, Wang WZ, *et al*. Stroke in China: epidemiology, prevention, and management strategies. *The Lancet Neurology* 2007; 6(5):456–464.
4. Liu L, Wang D, Wong KS, *et al*. Stroke and stroke care in China: huge burden, significant workload, and a national priority. *Stroke* 2011; 42:3651–3654.
5. Zhao D, Liu J, Wang W, *et al*. Epidemiological Transition of Stroke in China Twenty-One-Year Observational Study From the Sino-MONICA-Beijing Project. *Stroke* 2008; 39:1668–1674.
6. Wang J, Xiong X. Current situation and perspectives of clinical study in integrative medicine in China. *Evidence-Based Complementary and Alternative Medicine* 2012; 2012.
7. Wu HM, Tang JL, Lin XP, *et al*. Acupuncture for stroke rehabilitation. *Stroke* 2008; 39(2):517–518.
8. Mahoney FI. Functional evaluation: the Barthel index. *Maryland State Medical Journal* 1965; 14:61–65.
9. Collin C, Wade DT, Davies S, *et al*. The Barthel ADL Index: a reliability study. *Disability & Rehabilitation* 1988; 10(2):61–63.
10. Granger CV, Dewis LS, Peters NC, *et al*. Stroke rehabilitation: analysis of repeated Barthel index measures. *Archives of physical medicine and rehabilitation* 1979; 60(1):14–17.
11. Loewen SC, Anderson BA. Reliability of the modified motor assessment scale and the Barthel index. *Physical Therapy* 1988; 68(7):1077–1081.
12. Adams HP Jr, Davis PH, Leira EC, *et al*. Baseline NIH Stroke Scale score strongly predicts outcome after stroke: A report of the Trial of Org 10172 in Acute Stroke Treatment (TOAST). *Neurology* 1999; 53:126–131.

For peer review only

13. Goldstein LB, Samsa GP. Reliability of the National Institutes of Health Stroke Scale extension to non-neurologists in the context of a clinical trial. *Stroke* 1997; 28(2):307–310.

14. Sanford J, Moreland J, Swanson LR, *et al*. Reliability of the Fugl-Meyer assessment for testing motor performance in patients following stroke. *Physical therapy* 1993; 73(7):447–454.

15. Sullivan KJ, Tilson JK, Cen SY, *et al*. Fugl-Meyer assessment of sensorimotor function after stroke: Standardized training procedure for clinical practice and clinical trials. *Stroke* 2011; 42:427–432.

16. Smith T, Gildeh N, Holmes C. The Montreal Cognitive Assessment: validity and utility in a memory clinic setting. *Canadian Journal of Psychiatry* 2007; 52(5):329.

17. Nasreddine ZS, Phillips NA, Bédirian V, *et al*. The Montreal Cognitive Assessment, MoCA: a brief screening tool for mild cognitive impairment. *Journal of the American Geriatrics Society* 2005; 53(4):695–699.

18. Bose M, Shah P. Analyzing Post Stroke Depression (PSD) Levels in Stroke Patients Using Zung Self-Rating Depression Scale. *Indian Journal of Physiotherapy and Occupational Therapy-An International Journal* 2012; 6(4):187–190.

19. McIntyre RS, Konarski JZ, Mancini DA, *et al*. Measuring the severity of depression and remission in primary care: validation of the HAMD–7 scale. *Canadian Medical Association Journal* 2005;173(11):1327–1334.

20. Williams JBW, Kobak KA, Bech P, *et al*. The GRID-HAMD: standardization of the Hamilton depression rating scale. *International clinical psychopharmacology* 2008; 23(3):120–129.

21. Gosman-Hedström G, Claesson L, Klingenstierna U, *et al*. Effects of acupuncture treatment on daily life activities and quality of life a controlled, prospective, and randomized study of acute stroke patients. *Stroke* 1998; 29(10):2100–2108.

22. Park J, White AR, James MA, *et al*. Acupuncture for subacute stroke rehabilitation: a sham-controlled, subject-and assessor-blind, randomized trial. *Archives of internal medicine* 2005; 165(17):2026–2031.

For peer review only

23. Gong X, Sucher NJ. Stroke therapy in traditional Chinese medicine (TCM): prospects for drug discovery and development. *Trends in pharmacological sciences* 1999; 20(5):191–196.

24. Wu B, Liu M, Liu H, *et al*. Meta-analysis of traditional Chinese patent medicine for ischemic stroke. *Stroke* 2007; 38(6):1973–1979.

25. Junhua Z, Menniti-Ippolito F, Xiumei G, *et al*. Complex traditional Chinese medicine for post stroke motor dysfunction a systematic review. *Stroke* 2009; 40(8):2797–2804.

26. World Health Organization S: Acupuncture: Review and analysis of reports on controlled clinical trials. Geneva, Switzerland: *World Health Organization* 2003.

27. Zhang SH, Liu M, Asplund K, *et al.* Acupuncture for acute stroke. *Stroke* 2005; 36:2327–2328.

28. Sze FK, Wong E, Or KKH, *et al*. Does acupuncture improve motor recovery after stroke? A meta-analysis of randomized controlled trials. *Stroke* 2002; 33(11):2604–2619.

29. Fontanarosa PB, Lundberg GD. Complementary, alternative, unconventional, and integrative medicine: call for papers for the annual coordinated theme issues of the AMA journals. *JAMA* 1997; 278(23):2111–2112.

30. Maizes V, Rakel D, Niemiec C. Integrative medicine and patient-centered care. *Explore: The Journal of Science and Healing* 2009; 5(5):277–289.

31. Wang J, Xiong X. Current situation and perspectives of clinical study in integrative medicine in China. *Evidence-Based Complementary and Alternative Medicine* 2012; 2012.

For peer review only

Integrative Medicine for Sub-acute Stroke Rehabilitation: Study Protocol for a Multicenter, Randomized, Controlled Trial

Journal:	*BMJ Open*
Manuscript ID:	bmjopen-2014-007080.R1
Article Type:	Protocol
Date Submitted by the Author:	11-Nov-2014

Complete List of Authors:	Fang, Jianqiao; The Third Affiliated Hospital of Zhejiang Chinese Medical University, Department of Acupuncture; Zhejiang Chinese Medical University, The Third Clinical Medical College<br>Chen, Lifang; The Third Affiliated Hospital of Zhejiang Chinese Medical University, Department of Acupuncture<br>Chen, Luni; Zhejiang Chinese Medical University, The Third Clinical Medical College<br>Wang, Chao; Zhejiang Chinese Medical University, The Third Clinical Medical College<br>Keeler, Crystal; Five Branches University, Innovations to Wellness<br>Ma, Ruijie; The Third Affiliated Hospital of Zhejiang Cheinses Medical University, Department of Acupuncture<br>Xu, Shouyu; The Third Affiliated Hospital of Zhejiang Chinese Medical University, Department of Rehabilitation<br>Shen, Laihua; Jiaxing Hospital of Traditional Chinese Medicine, Department of Acupuncture & Encephalopathy<br>Bao, Yehua; Hangzhou Hospital of Traditional Chinese Medicine, Department of Acupuncture & Rehabilitation<br>Ji, Conghua; Zhejiang Provincial Hospital of Traditional Chinese Medicine, The Clinical Research Institute

**Primary Subject Heading**:	Complementary medicine
Secondary Subject Heading:	Complementary medicine
Keywords:	STROKE MEDICINE, COMPLEMENTARY MEDICINE, Rehabilitation medicine < INTERNAL MEDICINE

Integrative Medicine for Sub-acute Stroke Rehabilitation: Study Protocol for a Multicenter, Randomized, Controlled Trial

Jianqiao Fang,[1,2*] Lifang Chen,[1] Luni Chen,[2] Chao Wang,[2] Crystal Lynn Keeler,[3] Ruijie Ma,[1] Shouyu Xu,[4] Laihua Shen,[5] Yehua Bao,[6] Conghua Ji,[7]

Correspondence to
Professor Jianqiao Fang[1,2*];
fangjianqiao7532@163.com

Lifang Chen[1]
Email: clfang@163.com

Luni Chen[2]
Email: clnhz@126.com

Chao Wang[2]
wangchaozy1126@163.com

Crystal Lynn Keeler[3]
Email: Crystal.clk@gmail.com

Ruijie Ma[1]
Email: maria7878@sina.com

Shouyu Xu[4]
Email: overnightjo@msn.com

Laihua Shen[5]
Email: slh86ly@163.com

Yehua Bao[6]
Email: byh@hz.cn

Conghua Ji[7]
Email: jchi_2005@163.com

For peer review only

For peer review only - http://bmjopen.bmj.com/site/about/guidelines.xhtml

ABSTRACT

Introduction: Many stroke patients receive integrative medicine in China, which includes the basic treatment of Western medicine and routine rehabilitation, in conjunction with acupuncture and Chinese medicine. The question whether integrative medicine is efficacious for stroke rehabilitation is still controversial and very little research currently exists on the integrated approach for this condition. Consequently, we will conduct a multicenter, randomized, controlled, assessor-blinded clinical trial to assess the effectiveness of integrative medicine on stroke rehabilitation.

Methods and analysis: 360 participants recruited from three large Chinese medical hospitals in Zhejiang Province will be randomly divided into the integrative medicine rehabilitation (IMR) group and the conventional rehabilitation (CR) group in a 1:1 ratio. Participants in IMR group will receive acupuncture and Chinese herbs in addition to basic Western medicine and rehabilitation treatment. The CR group will not receive acupuncture and Chinese herbal medicine. The assessment data will be collected at baseline, 4 and 8 weeks post-randomization, and then at 12-weeks follow-up. The primary outcome assessment tool, the Modified Barthel Index (MBI), will be used to evaluate activities of daily living (ADL). The secondary outcomes will be measured by the National Institutes of Health Stroke Scale (NIHSS) for neurologic deficits, Fugl-Meyer Assessment (FMA) for motor dysfunction, and the Mini-Mental State Examination（MMSE）and Montreal Cognitive Assessment（MoCA）for cognitive impairment. For emotional disorders, the Hamilton’s Depression Scale (HAMD) and Self-Rating Depression Scale (SDS) will measure emotional state, and the incidence of adverse events (AEs) will also be observed.

Ethics and dissemination: Ethical approval was obtained from ethics committees of the Third Affiliated Hospital of Zhejiang Traditional Chinese Medical University, the Hangzhou Hospital of Traditional Chinese Medicine and the Jiaxing Hospital of Traditional Chinese Medicine. The results will be disseminated in a peer-reviewed journal and presented at international congresses. The results will also be

For peer review only

For peer review only - http://bmjopen.bmj.com/site/about/guidelines.xhtml

disseminated to patients by telephone, during follow-up calls inquiring on patient's post-study health status.

Trial registration: Chinese Clinical Trial Register: ChiCTR-TRC-12001972, http://www.chictr.org/en/proj/show.aspx?proj=2561

Keywords Integrative Medicine, Sub-acute Stroke, Study Protocol, Randomized Controlled Trial

For peer review only

INTRODUCTION

Stroke is the second most common cause of death and leading cause of adult disability worldwide[1], The number of patients who die from stroke is more than three times that from coronary heart disease[2]. Modern Western medicine in China undoubtedly occupies the dominant position in prevention and treatment of stroke. However, most patients with stroke are treated with one or more types of traditional Chinese medicine (TCM) in addition to Western medicine. The role of TCM should not be ignored[3-5]. With the development of integrative medicine, which was established in the 1980s, more and more stroke patients receive integrative medicine treatment. The main integrative treatment includes the standard Western medicine and rehabilitation for stroke, as well as acupuncture and (or) Chinese herbs[6]. Nevertheless, there is not yet enough evidence to show the effect of integrative medicine for stroke. More rigorously designed, large scale, multicenter randomized trials are necessary, to assess the effectiveness of integrative medicine on stroke rehabilitation[7].

METHODS

Study design

The study is a clinical research design on integrated rehabilitation with traditional Chinese and Western medicine on sub-acute stage of stroke, in a multicenter, randomized, controlled, assessor-blinded clinical trial. Participants recruited from three large Chinese medical hospitals, will be randomly divided into two groups (IMR group and CR group) using Excel generated random numbers list. The CR group will receive basic Western medical treatment and rehabilitation, which includes physical therapy treatment, and/or cognitive training for cognitive impairment, and/or psychological counseling for emotional disorders, six days per week. The IMR group will be additionally given acupuncture (thirty minutes of acupuncture therapy daily for six days per week lasting eight weeks) and Chinese herbs (treatment based on syndrome differentiation for once a day lasting eight weeks). The specific route diagram is presented in figure 1.

For peer review only - http://bmjopen.bmj.com/site/about/guidelines.xhtml

Participant recruitment

After getting the approval of Institutional Review Board, we recruited participants by advertising in local newspapers, health-related TV programs, Internet and posters in hospitals and communities. The recruiting time was from March 1, 2012 to December 31, 2014. The patients intending to join the study can consult with study coordinators regarding any questions they may have. Once the patients qualified and agreed to participate in the study, informed consent was obtained prior to running the series of baseline measurement assessments.

Inclusion criteria

To be eligible, participants must meet the following conditions: (1) Patients 35-80 years old, with a recent (30～40 days) ischemic stroke; (2) Patients with a NIHSS score between 4 and 24; (3) The stroke should be the first incidence or patients can have a history of stroke, but without disability (modified Rankin Scale, mRS score ≤1).

Exclusion criteria

Participants who conform to any conditions below will be excluded: (1) Patients received thrombolytic therapy or participated in other clinical trials in last three months; (2) Patients who suffered from serious heart, liver, kidney related diseases, blood coagulation dysfunction, or severe mental disorders; (3) Patients who can not accept acupuncture, and(or) Chinese medicine treatment; (4) Women who are pregnant or breast-feeding; (5) patients with congenital disabilities.

Ethical considerations

Each of the ethics committees of the Third Affiliated Hospital of Zhejiang Traditional Chinese Medical University, Hangzhou Hospital of Traditional Chinese Medicine and Jiaxing Hospital of Traditional Chinese Medicine, all approved the study. The purpose, nature, and potential risks of the experiments were fully explained to the patients and their families. All patients gave their written and oral informed consent before participating in the study.

Randomization and blinding

Randomization was done by Excel computer software in the study. The generated list

For peer review only - http://bmjopen.bmj.com/site/about/guidelines.xhtml

For peer review only

of random numbers was printed, cut into small pieces, separated and placed into sequentially numbered, opaque, sealed envelopes. The envelopes were saved by the special screeners. When the participant is included, the screeners opened the envelope to get the group information. Then the subject was informed whether they would be in the treatment group or control group, with or without acupuncture & Chinese herbs. All of the rehabilitation therapists, outcome assessors, and data analysts are blinded to group assignments. However, it is impossible to make acupuncturists blinded, because they are trained to perform the treatment for participants of IMR group.

Interventions and comparison

The study is a randomized clinical trial carried out in three centers. Participants will be randomized to either the IMR group or CR group. Both groups will receive conventional stroke rehabilitation care, which includes normal limb posture, physiotherapy (PT) and occupational therapy (OT), and/or cognitive training for cognitive impairment, and/or psychological counseling for emotional disorder. The rehabilitation team develops the rehabilitation program according to the investigator's brochure. Rehabilitation includes physiotherapy and occupational therapy for two hours per day, six days per week for each participant. The IMR group will receive 30 additional minutes of acupuncture therapy every day, six days per week and take Chinese herbal decoction (twice a day) for eight weeks during the inpatient stay.

Acupuncture treatment

The acupuncture program, developed by experts of our project group after many discussions, was performed by certified acupuncturists with more than five years of clinical experience. To ensure the same condition, all of the treatment protocols and processes are detailed below:

Scalp acupuncture: Select filiform needle (size 0.25 mm × 40 mm, Huatuo brand, manufactured by Suzhou Medical Appliance in Suzhou, Jiangsu Province China), swiftly insert the needle subcutaneously in 30° to the scalp on the top midline, the motor area and the sensory area of the affected side.

Body acupuncture (the affected side): LI15 (JianYu), LI11 (QuChi), LI10 (ShouSanLi), SJ5 (WaiGuan), LI4 (HeGu) for upper limbs; ST32 (BiGuan), ST36

For peer review only - http://bmjopen.bmj.com/site/about/guidelines.xhtml

(ZuSanLi), GB34 (YangLingQuan), GB39 (XuanZhong), BL60 (KunLun) for lower limbs. Acupoints of the above are referred to the People's Republic of China, State Standard Name and Location of Acupoints (GB 12346-2006).

Modification according to dysfunction after stroke: For cognitive impairment patients, add GV20 (BaiHui), GV24 (ShenTing), GB13 (BenShen), EX-HN1 (SiShenCong), Temple-Three-Needles (which is located in temple area, on the opposite side of hemiplegia, the first needle is located in 2 cun straight above ear apex, then the second and third needles are separately at the lateral 1 cun of the first needle). For emotional disorder patients, add LR3 (TaiChong), PC6 (NeiGuan), GV20 (BaiHui), GV29 (YinTang), GV24 (ShenTing).

Modification according to syndrome differentiation: For disturbance of wind-fire type, add LR2 (XingJian), LR3 (TaiChong), LR14 (QiMen); For phlegm-stasis blocking collaterals, add SP10 (XueHai), ST40 (FengLong); For yin deficiency and wind act, add SP6 (SanYinJiao), KI3 (TaiXi), LR3 (TaiChong); For qi deficiency and blood stasis type, add CV6 (QiHai), CV4 (GuanYuan), BL17 (GeShu).

Electro-acupuncture will be used when the patients De Qi (the sensation of aching, numbness, tingling or warmth). Then, LI15 (JianYu) and LI11 (QuChi), ST36 (ZuSanLi) and GB39 (XuanZhong) will be connected to GB6805-2 Electro-Acu Stimulators (Huayi Medical Supply & Equipment Co., Ltd, Shanghai, China). The parameter is 2Hz intermittent wave at the intensity within patients' tolerance.

Syndrome differentiation and Chinese herbal medicine

The prescription of Chinese herbs is based on syndrome differentiation. We formulated the treatment protocol through textbooks and ancient literatures, as well as experts' experiences, and the final version of the protocol was used for stroke patients of three centers before the trial carried out. There are four types according to syndrome differentiation: (1)For the syndrome of disturbance of wind-fire, Prescription: Tian Ma Gou Teng decoction modified (Tian Ma 9g, Gou Teng 15g, Shi Jue Ming 15g, Shan Zhi Zi 9g, Huang Qin 9g, Chuan Niu Xi 15g, Du Zhong 12g, Yi Mu Cao 15g, Sang Ji Sheng 15g,Ye Jiao Teng 9g, Fu Sheng 9g, raw Long Gu 30g, raw Mu Li 30g); (2)For the syndrome of phlegm-stasis blocking collaterals,

For peer review only - http://bmjopen.bmj.com/site/about/guidelines.xhtml

Prescription: Ban Xia Bai Zhu Tian Ma decoction and Tao Hong Si Wu decoction modified (Ban Xia 9g, Bai Zhu 9g, Tian Ma 9g, Fu Lin 9g, Ju Hong 6g, Sheng Di 15g, Dang Gui 15g, Chuan Xiong 9g, Tao Ren 9g, Hong Hua 6g) (3)For the syndrome of yin deficiency and wind act, Prescription: Zhen Gan Xi Feng decoction modified (raw Long Gu 15g, raw Mu Li 15g, Dai Zhe Shi 30g, Gui Ban 15g, Bai Shao 15g, Xuan Shen 15g, Tian Dong 15g, Chuan Lian Zi 6g, Yin Chen 6g, Chuan Xiong 15g, raw Mai Ya 6g, fried Gan Cao 6g) (4)For the syndrome of qi deficiency and blood stasis, Prescription: Bu Yang Huan Wu decoction modified (raw Huang Qi 30g, Dang Gui 15g, Tao Ren 6g, Hong Hua 6g, Di Long 12g, Chi Shao 15g).

Conventional Rehabilitation Group

Patients in CR group do not receive acupuncture and Chinese herbs. This group only receives basic Western medical and rehabilitation treatment, in the same frequency, with the same course of treatment as the IMR group.

Outcome assessment

The assessment data will be collected at baseline, 4 and 8 weeks post-randomization, and then 12 weeks after completing the treatment.

Baseline assessment

Demographic data includes gender, age, nationality, education level, occupation, and marital status. Information on stroke risk factors regarding smoking, drinking, height, weight, blood pressure, family history of stroke, blood lipids, and blood sugar is gathered through review of the medical records. Several classifications of disease data regarding: rehabilitation evaluation scales on neurological deficit, sensory motor, cognitive, and emotional disorders will be also analyzed before randomization.

Primary outcome measurement

The primary outcome measurement is the Modified Barthel Index (MBI), which was developed in 1955 as a simple index of independence useful in scoring disability[8]. The MBI scale is a reliable measure of functional independence. It is sensitive and valid to evaluate dependence in the activities of daily living (ADL). It includes ten variables (defecating, urinating, feeding, bathing, grooming, dressing, toileting, transfer, walking, and stairs) describing ADL and mobility. A higher number is

For peer review only - http://bmjopen.bmj.com/site/about/guidelines.xhtml

associated with a greater likelihood of being able to live at home with a degree of independence. This index is used as a standardized assessment on rehabilitation wards and also as standardized follow-up assessment to determine whether gains achieved by patients with stroke while hospitalized are maintained after discharge[9-11].

Secondary outcome measures

The National Institutes of Health Stroke Scale (NIHSS) for neurologic deficits

The National Institutes of Health Stroke Scale (NIHSS) is a graded neurological examination that assesses consciousness, best gaze, visual field, facial palsy, motor arm, motor leg, limb ataxia, sensory, best language, dysphagia, and neglect. The scale was developed for use in acute-stroke trials, and has since been widely used as a standard part of the assessment in clinical trials. Its scores range is from 0 to 42, with scores above 25 indicating very severe neurological impairment, scores of 5 to 24 suggesting moderately severe to severe impairment, and scores below 5 indicate mild impairment[12-13].

The Fugl-Meyer Assessment (FMA) scale for motor dysfunction

The FMA was developed as the first quantitative evaluative instrument for measuring sensorimotor stroke recovery, which includes items dealing with the shoulder, elbow, forearm, wrist, and hand in the upper extremity (UE, 66 points) and the hip, knee, and ankle in the lower extremity (LE, 34 points)[14].The motor domain has well-established reliability and validity as an indicator of motor impairment severity across different stroke recovery time points[15].

The Mini-Mental State Examination（MMSE）and Montreal Cognitive Assessment（MoCA）for cognitive impairment

Cognitive function is assessed by MMSE and MoCA scale. MMSE is a brief wide-range screening test with 30 aggregate scores, which is more suitable for uneducated or old populations. It assesses memory, orientation, calculation, attention, expressing, and reading. In fact, MMSE is widely used because of its high specificity, but it can’t subtly detect mild cognitive impairment (MCI) patients, whose scores are in the normal range[16]. In contrast, MoCA is more sensitive. It is also a test with 30 points, of which items including visual-spatial abilities, executive functions, attention,

For peer review only - http://bmjopen.bmj.com/site/about/guidelines.xhtml

concentration, memory, language, orientation. The MoCA detects MCI patients with 90% sensitivity and 87% specificity[17].

The Self-Rating Depression Scale (SDS) and Hamilton's Depression Scale (HAMD) for emotional disorder

SDS is a self-report instrument covering 20 items, either positive or negative, with a four-point scale ranging from 1 to 4. The standardized score is the total score times 1.25, which results in 25 to 100[18]. Furthermore, in antidepressant clinical trials, the Hamilton Depression Rating Scale has been the "gold standard" for use[19]. The HAMD Scale we selected includes 24 items, consisting of sense of guilt, sleeping problems, suicide, interest, anxiety, loss of weight, self-abasement, hopelessness, and so on, 14 items with a score of 0-4 and 10 items with score of 0-2. The range of scores for the whole test is 0-76. Higher scores indicate a higher level of mental disorder, respectively[20].

The incidence of adverse events

Participants are to be questioned and report all adverse events (AEs) at each visit point, and all AEs reports will be recorded and assessed by the investigators. If serious adverse events happen, the researchers should report to the principal investigator and ethics committee immediately, who will make a decision on whether the participant needs to withdraw from the study. If the participant suffered serious adverse events, unbinding is permissible, and procedure is followed for revealing a participant's allocated intervention during the trial. Compensation will be provided to those who suffer harm from trial participation.

In order to assess the safety of herbal medicine, we will perform the following tests on participants of IMR group at the baseline (week 0) and after treatment (week 12): blood routine test, urine routine test, feces routine test, kidney function test, liver function test. In addition, investigators will ask every subject at each visit whether they have experienced allergies or gastrointestinal discomfort during the study period.

The adverse events of acupuncture may include local bleeding, hematoma, pallor, sweating or dizziness, fainting during the acupuncture treatment, unbearable prickling, retained needle after treatment. The investigator should record the date of occurrence,

For peer review only - http://bmjopen.bmj.com/site/about/guidelines.xhtml

time, degree, measurement related to the treatment, and consequence.

Quality control and data management

This is a 20-week clinical trial, in which participants need to take herbal medicine and acupuncture for 8 weeks, and accept a 12-week follow-up, attend four assessment visits (rehabilitation evaluation), obtain one set of laboratory tests (safety assessments), Before the study, the trial protocol has been reviewed and revised by experts on acupuncture, neurology, rehabilitation, statistics, and methodology several times. All the members belonging to the trial are asked to take part in a series of training to ensure that the personnel involved fully understand the research protocol and standard operating procedures for the study. During the study, the Clinical Research Institute of Zhejiang Provincial is responsible for generating the allocation sequence, quality control, and censors have regular visits (once a month) to monitor protocol violations, the recruitment rate, AEs and participant compliance. This clinical trial is independent from sponsors and competing interests. The clinical coordinators of three centers are specifically designated to enroll participants, and assign participants to interventions, but not to participate in treatment and assessment for subjects. Outcome assessors and data analysts will be blinded after assignment to interventions, without access to patients' group information.

The measurements are mainly rehabilitation evaluations. All of the data will first be recorded on the paper version of the case report form (CRF) by assessors, then double entered into EDC system electronically. A specified statistics centre of the Clinical Research Institute of Zhejiang Province will responsible for data management. All data will be double entered to ensure accuracy. The source of any inconsistencies will be explored and resolved.

Sample size calculation

The primary efficacy parameter is the change in MBI scores from baseline to the end of treatment after eight weeks. Sample size calculations are based on our preliminary test and previous study[21-22], The expected difference between CR group and IMR group is 10 value, it is to say the score of MBI of IMR group was 10 value higher than

For peer review only - http://bmjopen.bmj.com/site/about/guidelines.xhtml

that of CR group, and the standard deviation is about 31, A two-sided 5% significance level and 80% power were considered, and the following equation was used:

$$n = \frac{2\left(z_{\alpha}/_{2} + z_{\beta}\right)^{2}\sigma^{2}}{\Delta^{2}}$$

Approximately 150 participants in each group were calculated to be required. Estimating a 20% dropout rate, each group will require 180 initial participants.

Statistical analysis

Efficacy and safety analyses will be conducted according to the intention-to-treat (ITT) principle by a statistician blinded to group allocation. Missing values will be imputed by the last-observation-carried-forward method. All statistical analyses will be performed using Statistical Product and Service Solutions (SPSS) statistical package program (ver. 17.0, SPSS Inc., Chicago, IL, USA). The primary outcomes (MBI) and FMA will undergo ITT analysis including all the patients who are randomized. The analysis of cognitive impairment, emotional disorder will be made among the defined population of corresponding dysfunction. Continuous variables will be expressed as means with standard deviations. For normally distributed variables, two independent samples will be compared by t-test. On the other hand, for abnormally distributed variables, Non-parametric tests will be used, and the data will be expressed as medians with ranges. A P-value of less than 0.05 is considered as statistical significance. Safety analysis is based on the frequency of adverse events relating to the treatment.

DISCUSSION

China's extensive clinical experience in the use of traditional Chinese medicines in stroke therapy indicates that TCM preparations are effective. More than 100 traditional medicines are currently in use for stroke therapy in China[23]. However, insufficient good-quality evidence on the effects of TCM in ischemic stroke exists on the primary outcome[24]. One possibility for lack of evidence in the literature could result from the significant clinical and methodological heterogeneity, preventing effective meta-analysis techniques. No meta-analysis have been performed and thus

For peer review only - http://bmjopen.bmj.com/site/about/guidelines.xhtml

no cumulative results obtained by pooling RCT data exist[25]. Further randomized controlled trials are justified.

Acupuncture is recommended for stroke according to the World Health Organization[26]. Literature reviews have demonstrated the safety of acupuncture, legitimizing its ethical use for patients, without causing harm[7]. Despite its safety, the limited availability of rigorous RCTs and the lack of research available on complementary and alternative medicine treatments like acupuncture, create a controversial opinion on its benefit for specific disease outcomes[7,27-28]. This lack of evidence necessitates additional RCT study on the clinical efficacy of integrative medicine on stroke outcomes.

Complementary or termed alternative medicine such as acupuncture and Chinese medicine, or integrative medicine has become increasingly prevalent and popular, not only in China, but also worldwide[29-30]. Integrated traditional Chinese and Western medicine for stroke rehabilitation is widely used in China, making it an ideal setting to study stroke treatment protocols. The integrated approach is forming some of China characteristics of stroke treatment modalities, which can be observed as a model for the rest of the world[31]. Many patients in China, with stroke, receive basic Western medicine and rehabilitation as well as acupuncture and Chinese medicine during hospital stays. So we are conducting this clinical trial, which is close to the actual treatment strategy in China, to objectively evaluate the clinical efficacy. Syndrome differentiation and treatment is the essence of Chinese medicine, so in our study, Chinese herbal prescriptions for stroke patients are clarified. Four types of herbal medicine are most common in clinic according to syndrome differentiation. The acupuncture program traditionally includes scalp acupuncture and body acupuncture, and in addition some acupoints are selected according to the patient's dysfunction and also syndrome differentiation. Under strict quality control, this study could potentially confirm whether or not integrative medicine (IM) is an effective adjunct to the standard rehabilitation therapy for ischemic stroke. Our study may also assess the efficacy of IM in promoting the recovery of motor dysfunction, cognitive impairment and emotional disorder.

CONCLUSION

For peer review only - http://bmjopen.bmj.com/site/about/guidelines.xhtml

This trial has been designed to provide robust data on the efficacy of integrative medicine for patients with ischemic stroke. It is also expected to clarify whether or not integrative medicine is effective for motor, cognitive, or emotional disorder after stroke.

Trial status

The trial is currently enrolling participants; recruitment started in March 1, 2012 and will be completed in December 31, 2014.

List of abbreviations used

FMA, Fugl-Meyer Assessment; MCI, Mild Cognitive Impairment; MMSE, Mini-Mental State Examination; MoCA, Montreal Cognitive Assessment; NIHSS, National Institutes of Health Stroke Scale; VFSS, Video-fluoroscopic Swallowing Study; SPSS, Statistical Product and Service Solutions; mRS, modified Rankin Scale; IMR, integrative medicine rehabilitation; CR, conventional rehabilitation; MBI, Modified Barthel Index; ADL, activities of daily living; HAMD, Hamilton's Depression Scale; SDS, Self-Rating Depression Scale; AEs, adverse events; TCM, traditional Chinese medicine; PT, physiotherapy; OT, occupational therapy; IM, integrative medicine.

Competing interests

The authors declare that they have no competing interests.

Authors' contributions

JQF, LFC, LNC, CW, CLK and CHJ and participated in the conception and design of the trial, planning the analysis of the data and drafting the manuscript. RJM, SYX, LHS, and YHB participated in data collection and are in charge of recruitment and treatment of patients in each center. All the authors discussed, revised and approved the final manuscript.

Authors' information

[1] Department of Acupuncture, The Third Affiliated Hospital of Zhejiang Traditional Chinese Medical University, No. 219 Moganshan Road, XiHu District, Hangzhou, Zhejiang Province 310005, China

For peer review only - http://bmjopen.bmj.com/site/about/guidelines.xhtml

[2] The Third Clinical Medical College of Zhejiang Traditional Chinese Medical University, No. 548 Binwen Road, Binjiang District, Hangzhou, Zhejiang Province 310053, China

[3] Innovations to Wellness. Affiliated with Five Branches University. 3031 Tisch Way, 5th floor Administration. San Jose, CA, 95128.

[4] Department of Rehabilitation, The Third Affiliated Hospital of Zhejiang Traditional Chinese Medical University, No. 219 Moganshan Road, XiHu District, Hangzhou, Zhejiang Province 310005, China

[5] Department of Acupuncture & Encephalopathy, Jiaxing Hospital of Traditional Chinese Medicine. No. 1501 Zhongshan East Road, Jiaxing, Zhejiang Province 310012, China

[6] Department of Acupuncture & Rehabilitation, Hangzhou Hospital of Traditional Chinese Medicine. No. 453 Tiyuchang Road, XiHu District, Hangzhou, Zhejiang Province 310007, China

[7] The Clinical Research Institute of Zhejiang Provincial Hospital of Traditional Chinese Medicine, No. 54 Youdian Road, Hangzhou, Zhejiang Province 310006, China

Acknowledgements and Funding

This study is funded by a project grant from the Provincial Administration of Traditional Chinese Medicine of Zhejiang (2011ZGG003), and the funder will not have a role in the conduction or interpretation of this trial, or in any decision to publish the results. The team would like to thank three postgraduate students who contributed their time to the preliminary experiments (Pei Luo, Wei Dong, and Lu Zhang). We also appreciate the help and effort from the people participating in this trial.

Provenance and peer review Not commissioned; peer reviewed for ethical and funding approval prior to submission.

Open Access This is an Open Access article distributed in accordance with the Creative Commons Attribution Non Commercial (CC BY-NC 3.0) license, which permits others to distribute, remix, adapt, build upon this work non-commercially, and license their derivative works on different terms, provided the original work is

For peer review only - http://bmjopen.bmj.com/site/about/guidelines.xhtml

properly cited and the use is non-commercial. See: http://creativecommons.org/licenses/by-nc/3.0/

References

1. Bonita R, Mendis S, Truelsen T, *et al*. The global stroke initiative. *The Lancet Neurology* 2004; 3(7):391–393.
2. Wang L, Kong L, Wu F, *et al*. Preventing chronic diseases in China. *The Lancet* 2005; 366(9499):1821–1824.
3. Liu M, Wu B, Wang WZ, *et al*. Stroke in China: epidemiology, prevention, and management strategies. *The Lancet Neurology* 2007; 6(5):456–464.
4. Liu L, Wang D, Wong KS, *et al*. Stroke and stroke care in China: huge burden, significant workload, and a national priority. *Stroke* 2011; 42:3651–3654.
5. Zhao D, Liu J, Wang W, *et al*. Epidemiological Transition of Stroke in China Twenty-One-Year Observational Study From the Sino-MONICA-Beijing Project. *Stroke* 2008; 39:1668–1674.
6. Wang J, Xiong X. Current situation and perspectives of clinical study in integrative medicine in China. *Evidence-Based Complementary and Alternative Medicine* 2012; 2012.
7. Wu HM, Tang JL, Lin XP, *et al*. Acupuncture for stroke rehabilitation. *Stroke* 2008; 39(2):517–518.
8. Mahoney FI. Functional evaluation: the Barthel index. *Maryland State Medical Journal* 1965; 14:61–65.
9. Collin C, Wade DT, Davies S, *et al*. The Barthel ADL Index: a reliability study. *Disability & Rehabilitation* 1988; 10(2):61–63.
10. Granger CV, Dewis LS, Peters NC, *et al*. Stroke rehabilitation: analysis of repeated Barthel index measures. *Archives of physical medicine and rehabilitation* 1979; 60(1):14–17.
11. Loewen SC, Anderson BA. Reliability of the modified motor assessment scale and the Barthel index. *Physical Therapy* 1988; 68(7):1077–1081.
12. Adams HP Jr, Davis PH, Leira EC, *et al*. Baseline NIH Stroke Scale score strongly

predicts outcome after stroke: A report of the Trial of Org 10172 in Acute Stroke Treatment (TOAST). *Neurology* 1999; 53:126–131.

13. Goldstein LB, Samsa GP. Reliability of the National Institutes of Health Stroke Scale extension to non-neurologists in the context of a clinical trial. *Stroke* 1997; 28(2):307–310.
14. Sanford J, Moreland J, Swanson LR, *et al*. Reliability of the Fugl-Meyer assessment for testing motor performance in patients following stroke. *Physical therapy* 1993; 73(7):447–454.
15. Sullivan KJ, Tilson JK, Cen SY, *et al*. Fugl-Meyer assessment of sensorimotor function after stroke: Standardized training procedure for clinical practice and clinical trials. *Stroke* 2011; 42:427–432.
16. Wind AW, Schellevis FG, Van Staveren G, *et al*. Limitations of the Mini−Mental State Examination in diagnosing dementia in general practice[J]. *International journal of geriatric psychiatry* 1997;12(1): 101-108.
17. Nasreddine ZS, Phillips NA, Bédirian V, *et al*. The Montreal Cognitive Assessment, MoCA: a brief screening tool for mild cognitive impairment. *Journal of the American Geriatrics Society* 2005; 53(4):695–699.
18. Bose M, Shah P. Analyzing Post Stroke Depression (PSD) Levels in Stroke Patients Using Zung Self-Rating Depression Scale. *Indian Journal of Physiotherapy and Occupational Therapy-An International Journal* 2012; 6(4):187–190.
19. McIntyre RS, Konarski JZ, Mancini DA, *et al*. Measuring the severity of depression and remission in primary care: validation of the HAMD–7 scale. *Canadian Medical Association Journal* 2005;173(11):1327–1334.
20. Williams JBW, Kobak KA, Bech P, *et al*. The GRID-HAMD: standardization of the Hamilton depression rating scale. *International clinical psychopharmacology* 2008; 23(3):120–129.
21. Gosman-Hedström G, Claesson L, Klingenstierna U, *et al*. Effects of acupuncture treatment on daily life activities and quality of life a controlled, prospective, and randomized study of acute stroke patients. *Stroke* 1998; 29(10):2100–2108.

For peer review only

22. Park J, White AR, James MA, *et al*. Acupuncture for subacute stroke rehabilitation: a sham-controlled, subject-and assessor-blind, randomized trial. *Archives of internal medicine* 2005; 165(17):2026–2031.

23. Gong X, Sucher NJ. Stroke therapy in traditional Chinese medicine (TCM): prospects for drug discovery and development. *Trends in pharmacological sciences* 1999; 20(5):191–196.

24. Wu B, Liu M, Liu H, *et al*. Meta-analysis of traditional Chinese patent medicine for ischemic stroke. *Stroke* 2007; 38(6):1973–1979.

25. Junhua Z, Menniti-Ippolito F, Xiumei G, *et al*. Complex traditional Chinese medicine for post stroke motor dysfunction a systematic review. *Stroke* 2009; 40(8):2797–2804.

26. World Health Organization S: Acupuncture: Review and analysis of reports on controlled clinical trials. Geneva, Switzerland: *World Health Organization* 2003.

27. Zhang SH, Liu M, Asplund K, *et al.* Acupuncture for acute stroke. *Stroke* 2005; 36:2327–2328.

28. Sze FK, Wong E, Or KKH, *et al*. Does acupuncture improve motor recovery after stroke? A meta-analysis of randomized controlled trials. *Stroke* 2002; 33(11):2604–2619.

29. Fontanarosa PB, Lundberg GD. Complementary, alternative, unconventional, and integrative medicine: call for papers for the annual coordinated theme issues of the AMA journals. *JAMA* 1997; 278(23):2111–2112.

30. Maizes V, Rakel D, Niemiec C. Integrative medicine and patient-centered care. *Explore: The Journal of Science and Healing* 2009; 5(5):277–289.

31. Wang J, Xiong X. Current situation and perspectives of clinical study in integrative medicine in China. *Evidence-Based Complementary and Alternative Medicine* 2012; 2012.

For peer review only - http://bmjopen.bmj.com/site/about/guidelines.xhtml

Integrative Medicine for Sub-acute Stroke Rehabilitation: Study Protocol for a Multicenter, Randomized, Controlled Trial

Jianqiao Fang,[1,2*] Lifang Chen,[1] Luni Chen,[2] Chao Wang,[2] Crystal Lynn Keeler,[3] Ruijie Ma,[1] Shouyu Xu,[4] Laihua Shen,[5] Yehua Bao,[6] Conghua Ji,[7]

Correspondence to
Professor Jianqiao Fang[1,2*];
fangjianqiao7532@163.com

Lifang Chen[1]
Email: clfang@163.com

Luni Chen[2]
Email: clnhz@126.com

Chao Wang[2]
wangchaozy1126@163.com

Crystal Lynn Keeler[3]
Email: Crystal.clk@gmail.com

Ruijie Ma[1]
Email: maria7878@sina.com

Shouyu Xu[4]
Email: overnightjo@msn.com

Laihua Shen[5]
Email: slh86ly@163.com

Yehua Bao[6]
Email: byh@hz.cn

Conghua Ji[7]
Email: jchi_2005@163.com

For peer review only

ABSTRACT

Introduction: Many stroke patients receive integrative medicine in China, which includes the basic treatment of Western medicine and routine rehabilitation, in conjunction with acupuncture and Chinese medicine. The question whether integrative medicine is efficacious for stroke rehabilitation is still controversial and very little research currently exists on the integrated approach for this condition. Consequently, we will conduct a multicenter, randomized, controlled, assessor-blinded clinical trial to assess the effectiveness of integrative medicine on stroke rehabilitation.

Methods and analysis: 360 participants recruited from three large Chinese medical hospitals in Zhejiang Province will be randomly divided into the integrative medicine rehabilitation (IMR) group and the conventional rehabilitation (CR) group in a 1:1 ratio. Participants in IMR group will receive acupuncture and Chinese herbs in addition to basic Western medicine and rehabilitation treatment. The CR group will not receive acupuncture and Chinese herbal medicine. The assessment data will be collected at baseline, 4 and 8 weeks post-randomization, and then at 12-weeks follow-up. The primary outcome assessment tool, the Modified Barthel Index (MBI), will be used to evaluate activities of daily living (ADL). The secondary outcomes will be measured by the National Institutes of Health Stroke Scale (NIHSS) for neurologic deficits, Fugl-Meyer Assessment (FMA) for motor dysfunction, and the Mini-Mental State Examination（MMSE）and Montreal Cognitive Assessment（MoCA）for cognitive impairment. For emotional disorders, the Hamilton's Depression Scale (HAMD) and Self-Rating Depression Scale (SDS) will measure emotional state, and the incidence of adverse events (AEs) will also be observed.

Ethics and dissemination: Ethical approval was obtained from ethics committees of the Third Affiliated Hospital of Zhejiang Traditional Chinese Medical University, the Hangzhou Hospital of Traditional Chinese Medicine and the Jiaxing Hospital of Traditional Chinese Medicine. The results will be disseminated in a peer-reviewed journal and presented at international congresses. The results will also be

For peer review only - http://bmjopen.bmj.com/site/about/guidelines.xhtml

disseminated to patients by telephone, during follow-up calls inquiring on patient's post-study health status.

Trial registration: Chinese Clinical Trial Register: ChiCTR-TRC-12001972, http://www.chictr.org/en/proj/show.aspx?proj=2561

Keywords Integrative Medicine, Sub-acute Stroke, Study Protocol, Randomized Controlled Trial

For peer review only

For peer review only - http://bmjopen.bmj.com/site/about/guidelines.xhtml

INTRODUCTION

Stroke is the second most common cause of death and leading cause of adult disability worldwide[1], The number of patients who die from stroke is more than three times that from coronary heart disease[2]. Modern Western medicine in China undoubtedly occupies the dominant position in prevention and treatment of stroke. However, most patients with stroke are treated with one or more types of traditional Chinese medicine (TCM) in addition to Western medicine. The role of TCM should not be ignored[3-5]. With the development of integrative medicine, which was established in the 1980s, more and more stroke patients receive integrative medicine treatment. The main integrative treatment includes the standard Western medicine and rehabilitation for stroke, as well as acupuncture and (or) Chinese herbs[6]. Nevertheless, there is not yet enough evidence to show the effect of integrative medicine for stroke. More rigorously designed, large scale, multicenter randomized trials are necessary, to assess the effectiveness of integrative medicine on stroke rehabilitation[7].

METHODS

Study design

The study is a clinical research design on integrated rehabilitation with traditional Chinese and Western medicine on sub-acute stage of stroke, in a multicenter, randomized, controlled, assessor-blinded clinical trial. Participants recruited from three large Chinese medical hospitals, will be randomly divided into two groups (IMR group and CR group) using Excel generated random numbers list. The CR group will receive basic Western medical treatment and rehabilitation, which includes physical therapy treatment, and/or cognitive training for cognitive impairment, and/or psychological counseling for emotional disorders, six days per week. The IMR group will be additionally given acupuncture (thirty minutes of acupuncture therapy daily for six days per week lasting eight weeks) and Chinese herbs (treatment based on syndrome differentiation for once a day lasting eight weeks). The specific route diagram is presented in figure 1.

For peer review only - http://bmjopen.bmj.com/site/about/guidelines.xhtml

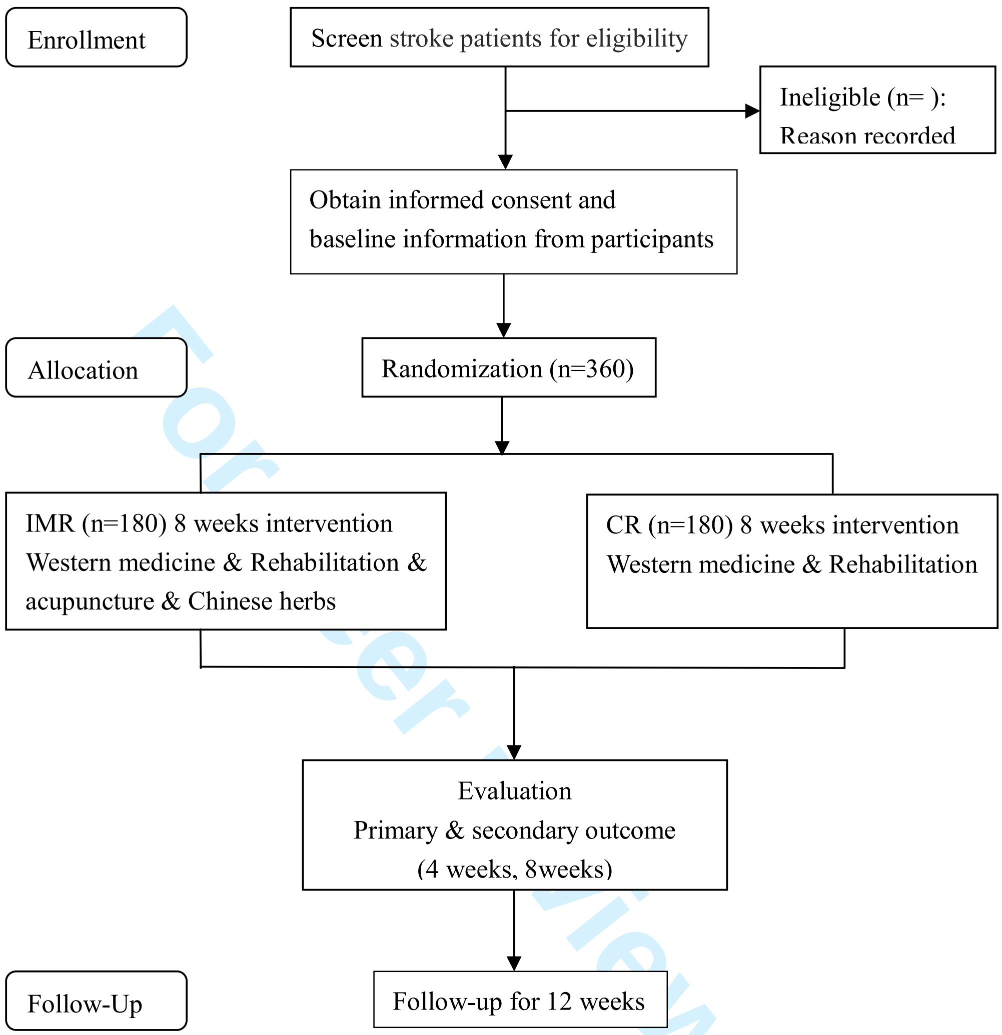

Figure 1.Route diagram of study design.

Participant recruitment

After getting the approval of Institutional Review Board, we recruited participants by advertising in local newspapers, health-related TV programs, Internet and posters in hospitals and communities. The recruiting time was from March 1, 2012 to December 31, 2014. The patients intending to join the study can consult with study coordinators regarding any questions they may have. Once the patients qualified and agreed to participate in the study, informed consent was obtained prior to running the series of baseline measurement assessments.

Inclusion criteria

To be eligible, participants must meet the following conditions: (1) Patients 35-80

For peer review only - http://bmjopen.bmj.com/site/about/guidelines.xhtml

years old, with a recent (30～40 days) ischemic stroke; (2) Patients with a NIHSS score between 4 and 24; (3) The stroke should be the first incidence or patients can have a history of stroke, but without disability (modified Rankin Scale, mRS score ≤1).

Exclusion criteria

Participants who conform to any conditions below will be excluded: (1) Patients received thrombolytic therapy or participated in other clinical trials in last three months; (2) Patients who suffered from serious heart, liver, kidney related diseases, blood coagulation dysfunction, or severe mental disorders; (3) Patients who can not accept acupuncture, and(or) Chinese medicine treatment; (4) Women who are pregnant or breast-feeding; (5) patients with congenital disabilities.

Ethical considerations

Each of the ethics committees of the Third Affiliated Hospital of Zhejiang Traditional Chinese Medical University, Hangzhou Hospital of Traditional Chinese Medicine and Jiaxing Hospital of Traditional Chinese Medicine, all approved the study. The purpose, nature, and potential risks of the experiments were fully explained to the patients and their families. All patients gave their written and oral informed consent before participating in the study.

Randomization and blinding

Randomization was done by Excel computer software in the study. The generated list of random numbers was printed, cut into small pieces, separated and placed into sequentially numbered, opaque, sealed envelopes. The envelopes were saved by the special screeners. When the participant is included, the screeners opened the envelope to get the group information. Then the subject was informed whether they would be in the treatment group or control group, with or without acupuncture & Chinese herbs. All of the rehabilitation therapists, outcome assessors, and data analysts are blinded to group assignments. However, it is impossible to make acupuncturists blinded, because they are trained to perform the treatment for participants of IMR group.

Interventions and comparison

For peer review only - http://bmjopen.bmj.com/site/about/guidelines.xhtml

The study is a randomized clinical trial carried out in three centers. Participants will be randomized to either the IMR group or CR group. Both groups will receive conventional stroke rehabilitation care, which includes normal limb posture, physiotherapy (PT) and occupational therapy (OT), and/or cognitive training for cognitive impairment, and/or psychological counseling for emotional disorder. The rehabilitation team develops the rehabilitation program according to the investigator's brochure. Rehabilitation includes physiotherapy and occupational therapy for two hours per day, six days per week for each participant. The IMR group will receive 30 additional minutes of acupuncture therapy every day, six days per week and take Chinese herbal decoction (twice a day) for eight weeks during the inpatient stay.

Acupuncture treatment

The acupuncture program, developed by experts of our project group after many discussions, was performed by certified acupuncturists with more than five years of clinical experience. To ensure the same condition, all of the treatment protocols and processes are detailed below:

Scalp acupuncture: Select filiform needle (size 0.25 mm × 40 mm, Huatuo brand, manufactured by Suzhou Medical Appliance in Suzhou, Jiangsu Province China), swiftly insert the needle subcutaneously in 30° to the scalp on the top midline, the motor area and the sensory area of the affected side.

Body acupuncture (the affected side): LI15 (JianYu), LI11 (QuChi), LI10 (ShouSanLi), SJ5 (WaiGuan), LI4 (HeGu) for upper limbs; ST32 (BiGuan), ST36 (ZuSanLi), GB34 (YangLingQuan), GB39 (XuanZhong), BL60 (KunLun) for lower limbs. Acupoints of the above are referred to the People's Republic of China, State Standard Name and Location of Acupoints (GB 12346-2006).

Modification according to dysfunction after stroke: For cognitive impairment patients, add GV20 (BaiHui), GV24 (ShenTing), GB13 (BenShen), EX-HN1 (SiShenCong), Temple-Three-Needles (which is located in temple area, on the opposite side of hemiplegia, the first needle is located in 2 cun straight above ear apex, then the second and third needles are separately at the lateral 1 cun of the first needle). For emotional disorder patients, add LR3 (TaiChong), PC6 (NeiGuan), GV20

For peer review only - http://bmjopen.bmj.com/site/about/guidelines.xhtml

(BaiHui), GV29 (YinTang), GV24 (ShenTing).

Modification according to syndrome differentiation: For disturbance of wind-fire type, add LR2 (XingJian), LR3 (TaiChong), LR14 (QiMen); For phlegm-stasis blocking collaterals, add SP10 (XueHai), ST40 (FengLong); For yin deficiency and wind act, add SP6 (SanYinJiao), KI3 (TaiXi), LR3 (TaiChong); For qi deficiency and blood stasis type, add CV6 (QiHai), CV4 (GuanYuan), BL17 (GeShu).

Electro-acupuncture will be used when the patients De Qi (the sensation of aching, numbness, tingling or warmth). Then, LI15 (JianYu) and LI11 (QuChi), ST36 (ZuSanLi) and GB39 (XuanZhong) will be connected to GB6805-2 Electro-Acu Stimulators (Huayi Medical Supply & Equipment Co., Ltd, Shanghai, China). The parameter is 2Hz intermittent wave at the intensity within patients' tolerance.

Syndrome differentiation and Chinese herbal medicine

The prescription of Chinese herbs is based on syndrome differentiation. We formulated the treatment protocol through textbooks and ancient literatures, as well as experts' experiences, and the final version of the protocol was used for stroke patients of three centers before the trial carried out. There are four types according to syndrome differentiation: (1)For the syndrome of disturbance of wind-fire, Prescription: Tian Ma Gou Teng decoction modified (Tian Ma 9g, Gou Teng 15g, Shi Jue Ming 15g, Shan Zhi Zi 9g, Huang Qin 9g, Chuan Niu Xi 15g, Du Zhong 12g, Yi Mu Cao 15g, Sang Ji Sheng 15g,Ye Jiao Teng 9g, Fu Sheng 9g, raw Long Gu 30g, raw Mu Li 30g); (2)For the syndrome of phlegm-stasis blocking collaterals, Prescription: Ban Xia Bai Zhu Tian Ma decoction and Tao Hong Si Wu decoction modified (Ban Xia 9g, Bai Zhu 9g, Tian Ma 9g, Fu Lin 9g, Ju Hong 6g, Sheng Di 15g, Dang Gui 15g, Chuan Xiong 9g, Tao Ren 9g, Hong Hua 6g) (3)For the syndrome of yin deficiency and wind act, Prescription: Zhen Gan Xi Feng decoction modified (raw Long Gu 15g, raw Mu Li 15g, Dai Zhe Shi 30g, Gui Ban 15g, Bai Shao 15g, Xuan Shen 15g, Tian Dong 15g, Chuan Lian Zi 6g, Yin Chen 6g, Chuan Xiong 15g, raw Mai Ya 6g, fried Gan Cao 6g) (4)For the syndrome of qi deficiency and blood stasis, Prescription: Bu Yang Huan Wu decoction modified (raw Huang Qi 30g, Dang Gui 15g, Tao Ren 6g, Hong Hua 6g, Di Long 12g, Chi Shao 15g).

For peer review only - http://bmjopen.bmj.com/site/about/guidelines.xhtml

Conventional Rehabilitation Group

Patients in CR group do not receive acupuncture and Chinese herbs. This group only receives basic Western medical and rehabilitation treatment, in the same frequency, with the same course of treatment as the IMR group.

Outcome assessment

The assessment data will be collected at baseline, 4 and 8 weeks post-randomization, and then 12 weeks after completing the treatment.

Baseline assessment

Demographic data includes gender, age, nationality, education level, occupation, and marital status. Information on stroke risk factors regarding smoking, drinking, height, weight, blood pressure, family history of stroke, blood lipids, and blood sugar is gathered through review of the medical records. Several classifications of disease data regarding: rehabilitation evaluation scales on neurological deficit, sensory motor, cognitive, and emotional disorders will be also analyzed before randomization.

Primary outcome measurement

The primary outcome measurement is the Modified Barthel Index (MBI), which was developed in 1955 as a simple index of independence useful in scoring disability[8]. The MBI scale is a reliable measure of functional independence. It is sensitive and valid to evaluate dependence in the activities of daily living (ADL). It includes ten variables (defecating, urinating, feeding, bathing, grooming, dressing, toileting, transfer, walking, and stairs) describing ADL and mobility. A higher number is associated with a greater likelihood of being able to live at home with a degree of independence. This index is used as a standardized assessment on rehabilitation wards and also as standardized follow-up assessment to determine whether gains achieved by patients with stroke while hospitalized are maintained after discharge[9-11].

Secondary outcome measures

The National Institutes of Health Stroke Scale (NIHSS) for neurologic deficits

The National Institutes of Health Stroke Scale (NIHSS) is a graded neurological examination that assesses consciousness, best gaze, visual field, facial palsy, motor arm, motor leg, limb ataxia, sensory, best language, dysphagia, and neglect. The scale

For peer review only - http://bmjopen.bmj.com/site/about/guidelines.xhtml

was developed for use in acute-stroke trials, and has since been widely used as a standard part of the assessment in clinical trials. Its scores range is from 0 to 42, with scores above 25 indicating very severe neurological impairment, scores of 5 to 24 suggesting moderately severe to severe impairment, and scores below 5 indicate mild impairment[12-13].

The Fugl-Meyer Assessment (FMA) scale for motor dysfunction

The FMA was developed as the first quantitative evaluative instrument for measuring sensorimotor stroke recovery, which includes items dealing with the shoulder, elbow, forearm, wrist, and hand in the upper extremity (UE, 66 points) and the hip, knee, and ankle in the lower extremity (LE, 34 points)[14].The motor domain has well-established reliability and validity as an indicator of motor impairment severity across different stroke recovery time points[15].

The Mini-Mental State Examination（MMSE） and Montreal Cognitive Assessment（MoCA）for cognitive impairment

Cognitive function is assessed by MMSE and MoCA scale. MMSE is a brief wide-range screening test with 30 aggregate scores, which is more suitable for uneducated or old populations. It assesses memory, orientation, calculation, attention, expressing, and reading. In fact, MMSE is widely used because of its high specificity, but it can't subtly detect mild cognitive impairment (MCI) patients, whose scores are in the normal range[16]. In contrast, MoCA is more sensitive. It is also a test with 30 points, of which items including visual-spatial abilities, executive functions, attention, concentration, memory, language, orientation. The MoCA detects MCI patients with 90% sensitivity and 87% specificity[17].

The Self-Rating Depression Scale (SDS) and Hamilton's Depression Scale (HAMD) for emotional disorder

SDS is a self-report instrument covering 20 items, either positive or negative, with a four-point scale ranging from 1 to 4. The standardized score is the total score times 1.25, which results in 25 to 100[18]. Furthermore, in antidepressant clinical trials, the Hamilton Depression Rating Scale has been the "gold standard" for use[19]. The HAMD Scale we selected includes 24 items, consisting of sense of guilt, sleeping

For peer review only - http://bmjopen.bmj.com/site/about/guidelines.xhtml

problems, suicide, interest, anxiety, loss of weight, self-abasement, hopelessness, and so on, 14 items with a score of 0-4 and 10 items with score of 0-2. The range of scores for the whole test is 0-76. Higher scores indicate a higher level of mental disorder, respectively[20].

The incidence of adverse events

Participants are to be questioned and report all adverse events (AEs) at each visit point, and all AEs reports will be recorded and assessed by the investigators. If serious adverse events happen, the researchers should report to the principal investigator and ethics committee immediately, who will make a decision on whether the participant needs to withdraw from the study. If the participant suffered serious adverse events, unbinding is permissible, and procedure is followed for revealing a participant's allocated intervention during the trial. Compensation will be provided to those who suffer harm from trial participation.

In order to assess the safety of herbal medicine, we will perform the following tests on participants of IMR group at the baseline (week 0) and after treatment (week 12): blood routine test, urine routine test, feces routine test, kidney function test, liver function test. In addition, investigators will ask every subject at each visit whether they have experienced allergies or gastrointestinal discomfort during the study period.

The adverse events of acupuncture may include local bleeding, hematoma, pallor, sweating or dizziness, fainting during the acupuncture treatment, unbearable prickling, retained needle after treatment. The investigator should record the date of occurrence, time, degree, measurement related to the treatment, and consequence.

Quality control and data management

This is a 20-week clinical trial, in which participants need to take herbal medicine and acupuncture for 8 weeks, and accept a 12-week follow-up, attend four assessment visits (rehabilitation evaluation), obtain one set of laboratory tests (safety assessments), Before the study, the trial protocol has been reviewed and revised by experts on acupuncture, neurology, rehabilitation, statistics, and methodology several times. All the members belonging to the trial are asked to take part in a series of training to ensure that the personnel involved fully understand the research protocol

For peer review only - http://bmjopen.bmj.com/site/about/guidelines.xhtml

and standard operating procedures for the study. During the study, the Clinical Research Institute of Zhejiang Provincial is responsible for generating the allocation sequence, quality control, and censors have regular visits (once a month) to monitor protocol violations, the recruitment rate, AEs and participant compliance. This clinical trial is independent from sponsors and competing interests. The clinical coordinators of three centers are specifically designated to enroll participants, and assign participants to interventions, but not to participate in treatment and assessment for subjects. Outcome assessors and data analysts will be blinded after assignment to interventions, without access to patients' group information.

The measurements are mainly rehabilitation evaluations. All of the data will first be recorded on the paper version of the case report form (CRF) by assessors, then double entered into EDC system electronically. A specified statistics centre of the Clinical Research Institute of Zhejiang Province will responsible for data management. All data will be double entered to ensure accuracy. The source of any inconsistencies will be explored and resolved.

Sample size calculation

The primary efficacy parameter is the change in MBI scores from baseline to the end of treatment after eight weeks. Sample size calculations are based on our preliminary test and previous study[21-22], The expected difference between CR group and IMR group is 10 value, it is to say the score of MBI of IMR group was 10 value higher than that of CR group, and the standard deviation is about 31, A two-sided 5% significance level and 80% power were considered, and the following equation was used:

$$n = \frac{2\left(z_{\alpha}/2 + z_{\beta} \right)^{2} \sigma^{2}}{\Delta^{2}}$$

Approximately 150 participants in each group were calculated to be required. Estimating a 20% dropout rate, each group will require 180 initial participants.

Statistical analysis

Efficacy and safety analyses will be conducted according to the intention-to-treat (ITT) principle by a statistician blinded to group allocation. Missing values will be imputed

For peer review only - http://bmjopen.bmj.com/site/about/guidelines.xhtml

by the last-observation-carried-forward method. All statistical analyses will be performed using Statistical Product and Service Solutions (SPSS) statistical package program (ver. 17.0, SPSS Inc., Chicago, IL, USA). The primary outcomes (MBI) and FMA will undergo ITT analysis including all the patients who are randomized. The analysis of cognitive impairment, emotional disorder will be made among the defined population of corresponding dysfunction. Continuous variables will be expressed as means with standard deviations. For normally distributed variables, two independent samples will be compared by t-test. On the other hand, for abnormally distributed variables, Non-parametric tests will be used, and the data will be expressed as medians with ranges. A P-value of less than 0.05 is considered as statistical significance. Safety analysis is based on the frequency of adverse events relating to the treatment.

DISCUSSION

China's extensive clinical experience in the use of traditional Chinese medicines in stroke therapy indicates that TCM preparations are effective. More than 100 traditional medicines are currently in use for stroke therapy in China[23]. However, insufficient good-quality evidence on the effects of TCM in ischemic stroke exists on the primary outcome[24]. One possibility for lack of evidence in the literature could result from the significant clinical and methodological heterogeneity, preventing effective meta-analysis techniques. No meta-analysis have been performed and thus no cumulative results obtained by pooling RCT data exist[25]. Further randomized controlled trials are justified.

Acupuncture is recommended for stroke according to the World Health Organization[26]. Literature reviews have demonstrated the safety of acupuncture, legitimizing its ethical use for patients, without causing harm[7]. Despite its safety, the limited availability of rigorous RCTs and the lack of research available on complementary and alternative medicine treatments like acupuncture, create a controversial opinion on its benefit for specific disease outcomes[7,27-28]. This lack of evidence necessitates additional RCT study on the clinical efficacy of integrative medicine on stroke outcomes.

For peer review only - http://bmjopen.bmj.com/site/about/guidelines.xhtml

Complementary or termed alternative medicine such as acupuncture and Chinese medicine, or integrative medicine has become increasingly prevalent and popular, not only in China, but also worldwide[29-30]. Integrated traditional Chinese and Western medicine for stroke rehabilitation is widely used in China, making it an ideal setting to study stroke treatment protocols. The integrated approach is forming some of China characteristics of stroke treatment modalities, which can be observed as a model for the rest of the world[31]. Many patients in China, with stroke, receive basic Western medicine and rehabilitation as well as acupuncture and Chinese medicine during hospital stays. So we are conducting this clinical trial, which is close to the actual treatment strategy in China, to objectively evaluate the clinical efficacy. Syndrome differentiation and treatment is the essence of Chinese medicine, so in our study, Chinese herbal prescriptions for stroke patients are clarified. Four types of herbal medicine are most common in clinic according to syndrome differentiation. The acupuncture program traditionally includes scalp acupuncture and body acupuncture, and in addition some acupoints are selected according to the patient's dysfunction and also syndrome differentiation. Under strict quality control, this study could potentially confirm whether or not integrative medicine (IM) is an effective adjunct to the standard rehabilitation therapy for ischemic stroke. Our study may also assess the efficacy of IM in promoting the recovery of motor dysfunction, cognitive impairment and emotional disorder.

CONCLUSION

This trial has been designed to provide robust data on the efficacy of integrative medicine for patients with ischemic stroke. It is also expected to clarify whether or not integrative medicine is effective for motor, cognitive, or emotional disorder after stroke.

Trial status

The trial is currently enrolling participants; recruitment started in March 1, 2012 and will be completed in December 31, 2014.

List of abbreviations used

FMA, Fugl-Meyer Assessment; MCI, Mild Cognitive Impairment; MMSE,

For peer review only - http://bmjopen.bmj.com/site/about/guidelines.xhtml

Mini-Mental State Examination; MoCA, Montreal Cognitive Assessment; NIHSS, National Institutes of Health Stroke Scale; VFSS, Video-fluoroscopic Swallowing Study; SPSS, Statistical Product and Service Solutions; mRS, modified Rankin Scale; IMR, integrative medicine rehabilitation; CR, conventional rehabilitation; MBI, Modified Barthel Index; ADL, activities of daily living; HAMD, Hamilton's Depression Scale; SDS, Self-Rating Depression Scale; AEs, adverse events; TCM, traditional Chinese medicine; PT, physiotherapy; OT, occupational therapy; IM, integrative medicine.

Competing interests

The authors declare that they have no competing interests.

Authors' contributions

JQF, LFC, LNC, CW, CLK and CHJ and participated in the conception and design of the trial, planning the analysis of the data and drafting the manuscript. RJM, SYX, LHS, and YHB participated in data collection and are in charge of recruitment and treatment of patients in each center. All the authors discussed, revised and approved the final manuscript.

Authors' information

[1] Department of Acupuncture, The Third Affiliated Hospital of Zhejiang Traditional Chinese Medical University, No. 219 Moganshan Road, XiHu District, Hangzhou, Zhejiang Province 310005, China

[2] The Third Clinical Medical College of Zhejiang Traditional Chinese Medical University, No. 548 Binwen Road, Binjiang District, Hangzhou, Zhejiang Province 310053, China

[3] Innovations to Wellness. Affiliated with Five Branches University. 3031 Tisch Way, 5th floor Administration. San Jose, CA, 95128.

[4] Department of Rehabilitation, The Third Affiliated Hospital of Zhejiang Traditional Chinese Medical University, No. 219 Moganshan Road, XiHu District, Hangzhou, Zhejiang Province 310005, China

For peer review only - http://bmjopen.bmj.com/site/about/guidelines.xhtml

[5] Department of Acupuncture & Encephalopathy, Jiaxing Hospital of Traditional Chinese Medicine. No. 1501 Zhongshan East Road, Jiaxing, Zhejiang Province 310012, China

[6] Department of Acupuncture & Rehabilitation, Hangzhou Hospital of Traditional Chinese Medicine. No. 453 Tiyuchang Road, XiHu District, Hangzhou, Zhejiang Province 310007, China

[7] The Clinical Research Institute of Zhejiang Provincial Hospital of Traditional Chinese Medicine, No. 54 Youdian Road, Hangzhou, Zhejiang Province 310006, China

Acknowledgements and Funding

This study is funded by a project grant from the Provincial Administration of Traditional Chinese Medicine of Zhejiang (2011ZGG003), and the funder will not have a role in the conduction or interpretation of this trial, or in any decision to publish the results. The team would like to thank three postgraduate students who contributed their time to the preliminary experiments (Pei Luo, Wei Dong, and Lu Zhang). We also appreciate the help and effort from the people participating in this trial.

Provenance and peer review Not commissioned; peer reviewed for ethical and funding approval prior to submission.

Open Access This is an Open Access article distributed in accordance with the Creative Commons Attribution Non Commercial (CC BY-NC 3.0) license, which permits others to distribute, remix, adapt, build upon this work non-commercially, and license their derivative works on different terms, provided the original work is properly cited and the use is non-commercial. See: http://creativecommons.org/licenses/by-nc/3.0/

References

1. Bonita R, Mendis S, Truelsen T, *et al*. The global stroke initiative. *The Lancet Neurology* 2004; 3(7):391–393.
2. Wang L, Kong L, Wu F, *et al*. Preventing chronic diseases in China. *The Lancet* 2005; 366(9499):1821–1824.

For peer review only - http://bmjopen.bmj.com/site/about/guidelines.xhtml

3. Liu M, Wu B, Wang WZ, *et al*. Stroke in China: epidemiology, prevention, and management strategies. *The Lancet Neurology* 2007; 6(5):456–464.

4. Liu L, Wang D, Wong KS, *et al*. Stroke and stroke care in China: huge burden, significant workload, and a national priority. *Stroke* 2011; 42:3651–3654.

5. Zhao D, Liu J, Wang W, *et al*. Epidemiological Transition of Stroke in China Twenty-One-Year Observational Study From the Sino-MONICA-Beijing Project. *Stroke* 2008; 39:1668–1674.

6. Wang J, Xiong X. Current situation and perspectives of clinical study in integrative medicine in China. *Evidence-Based Complementary and Alternative Medicine* 2012; 2012.

7. Wu HM, Tang JL, Lin XP, *et al*. Acupuncture for stroke rehabilitation. *Stroke* 2008; 39(2):517–518.

8. Mahoney FI. Functional evaluation: the Barthel index. *Maryland State Medical Journal* 1965; 14:61–65.

9. Collin C, Wade DT, Davies S, *et al*. The Barthel ADL Index: a reliability study. *Disability & Rehabilitation* 1988; 10(2):61–63.

10. Granger CV, Dewis LS, Peters NC, *et al*. Stroke rehabilitation: analysis of repeated Barthel index measures. *Archives of physical medicine and rehabilitation* 1979; 60(1):14–17.

11. Loewen SC, Anderson BA. Reliability of the modified motor assessment scale and the Barthel index. *Physical Therapy* 1988; 68(7):1077–1081.

12. Adams HP Jr, Davis PH, Leira EC, *et al*. Baseline NIH Stroke Scale score strongly predicts outcome after stroke: A report of the Trial of Org 10172 in Acute Stroke Treatment (TOAST). *Neurology* 1999; 53:126–131.

13. Goldstein LB, Samsa GP. Reliability of the National Institutes of Health Stroke Scale extension to non-neurologists in the context of a clinical trial. *Stroke* 1997; 28(2):307–310.

14. Sanford J, Moreland J, Swanson LR, *et al*. Reliability of the Fugl-Meyer assessment for testing motor performance in patients following stroke. *Physical therapy* 1993; 73(7):447–454.

For peer review only - http://bmjopen.bmj.com/site/about/guidelines.xhtml

15. Sullivan KJ, Tilson JK, Cen SY, *et al*. Fugl-Meyer assessment of sensorimotor function after stroke: Standardized training procedure for clinical practice and clinical trials. *Stroke* 2011; 42:427–432.

16. Wind AW, Schellevis FG, Van Staveren G, *et al*. Limitations of the Mini−Mental State Examination in diagnosing dementia in general practice[J]. *International journal of geriatric psychiatry* 1997;12(1): 101-108.

17. Nasreddine ZS, Phillips NA, Bédirian V, *et al*. The Montreal Cognitive Assessment, MoCA: a brief screening tool for mild cognitive impairment. *Journal of the American Geriatrics Society* 2005; 53(4):695–699.

18. Bose M, Shah P. Analyzing Post Stroke Depression (PSD) Levels in Stroke Patients Using Zung Self-Rating Depression Scale. *Indian Journal of Physiotherapy and Occupational Therapy-An International Journal* 2012; 6(4):187–190.

19. McIntyre RS, Konarski JZ, Mancini DA, *et al*. Measuring the severity of depression and remission in primary care: validation of the HAMD–7 scale. *Canadian Medical Association Journal* 2005;173(11):1327–1334.

20. Williams JBW, Kobak KA, Bech P, *et al*. The GRID-HAMD: standardization of the Hamilton depression rating scale. *International clinical psychopharmacology* 2008; 23(3):120–129.

21. Gosman-Hedström G, Claesson L, Klingenstierna U, *et al*. Effects of acupuncture treatment on daily life activities and quality of life a controlled, prospective, and randomized study of acute stroke patients. *Stroke* 1998; 29(10):2100–2108.

22. Park J, White AR, James MA, *et al*. Acupuncture for subacute stroke rehabilitation: a sham-controlled, subject-and assessor-blind, randomized trial. *Archives of internal medicine* 2005; 165(17):2026–2031.

23. Gong X, Sucher NJ. Stroke therapy in traditional Chinese medicine (TCM): prospects for drug discovery and development. *Trends in pharmacological sciences* 1999; 20(5):191–196.

24. Wu B, Liu M, Liu H, *et al*. Meta-analysis of traditional Chinese patent medicine for ischemic stroke. *Stroke* 2007; 38(6):1973–1979.

For peer review only - http://bmjopen.bmj.com/site/about/guidelines.xhtml

25. Junhua Z, Menniti-Ippolito F, Xiumei G, *et al*. Complex traditional Chinese medicine for post stroke motor dysfunction a systematic review. *Stroke* 2009; 40(8):2797–2804.
26. World Health Organization S: Acupuncture: Review and analysis of reports on controlled clinical trials. Geneva, Switzerland: *World Health Organization* 2003.
27. Zhang SH, Liu M, Asplund K, *et al.* Acupuncture for acute stroke. *Stroke* 2005; 36:2327–2328.
28. Sze FK, Wong E, Or KKH, *et al*. Does acupuncture improve motor recovery after stroke? A meta-analysis of randomized controlled trials. *Stroke* 2002; 33(11):2604–2619.
29. Fontanarosa PB, Lundberg GD. Complementary, alternative, unconventional, and integrative medicine: call for papers for the annual coordinated theme issues of the AMA journals. *JAMA* 1997; 278(23):2111–2112.
30. Maizes V, Rakel D, Niemiec C. Integrative medicine and patient-centered care. *Explore: The Journal of Science and Healing* 2009; 5(5):277–289.
31. Wang J, Xiong X. Current situation and perspectives of clinical study in integrative medicine in China. *Evidence-Based Complementary and Alternative Medicine* 2012; 2012.

For peer review only - http://bmjopen.bmj.com/site/about/guidelines.xhtml

SPIRIT — STANDARD PROTOCOL ITEMS: RECOMMENDATIONS FOR INTERVENTIONAL TRIALS

SPIRIT 2013 Checklist: Recommended items to address in a clinical trial protocol and related documents*

Section/item	Item No	Description
**Administrative information**		
Title	1	Descriptive title identifying the study design, population, interventions, and, if applicable, trial acronym **[page 1]**
Trial registration	2a	Trial identifier and registry name. If not yet registered, name of intended registry **[page 3]**
	2b	All items from the World Health Organization Trial Registration Data Set [page 3. See detail in URL http://www.chictr.org/en/proj/show.aspx?proj=2561]
Protocol version	3	Date and version identifier [Date: Jun 15,2012 ;Version identifier:4.0]
Funding	4	Sources and types of financial, material, and other support [page 15]
Roles and responsibilities	5a	Names, affiliations, and roles of protocol contributors [pages 14-15]
	5b	Name and contact information for the trial sponsor [page 15]
	5c	Role of study sponsor and funders, if any, in study design; collection, management, analysis, and interpretation of data; writing of the report; and the decision to submit the report for publication, including whether they will have ultimate authority over any of these activities [page 15]
	5d	Composition, roles, and responsibilities of the coordinating centre, steering committee, endpoint adjudication committee, data management team, and other individuals or groups overseeing the trial, if applicable (see Item 21a for data monitoring committee) [page 11]
**Introduction**		
Background and rationale	6a	Description of research question and justification for undertaking the l, including summary of relevant studies (published and unpublished) amining benefits and harms for each intervention [pages 2-4]
	6b	Explanation for choice of comparators [pages 2-4]

For peer review only - http://bmjopen.bmj.com/site/about/guidelines.xhtml

Objectives	7	Specific objectives or hypotheses [pages 2-4]
Trial design	8	Description of trial design including type of trial (eg, parallel group, ssover, factorial, single group), allocation ratio, and framework (eg, periority, equivalence, noninferiority, exploratory) [pages 4-5]

Methods: Participants, interventions, and outcomes

Study setting	9	Description of study settings (eg, community clinic, academic hospital) and list of countries where data will be collected. Reference to where list of study sites can be obtained [pages 6]
Eligibility criteria	10	Inclusion and exclusion criteria for participants. If applicable, eligibility criteria for study centres and individuals who will perform the interventions (eg, surgeons, psychotherapists) [pages 6]
Interventions	11a	Interventions for each group with sufficient detail to allow replication, including how and when they will be administered [pages 7-9]
	11b	Criteria for discontinuing or modifying allocated interventions for a given trial participant (eg, drug dose change in response to harms, participant request, or improving/worsening disease) [page11]
	11c	Strategies to improve adherence to intervention protocols, and any procedures for monitoring adherence (eg, drug tablet return, laboratory tests) [pages 11-12]
	11d	Relevant concomitant care and interventions that are permitted or prohibited during the trial [pages 11-12]
Outcomes	12	Primary, secondary, and other outcomes, including the specific measurement variable (eg, systolic blood pressure), analysis metric (eg, change from baseline, final value, time to event), method of aggregation (eg, median, proportion), and time point for each outcome. Explanation of the clinical relevance of chosen efficacy and harm outcomes is strongly recommended [pages 9-11]
Participant timeline	13	Time schedule of enrolment, interventions (including any run-ins and washouts), assessments, and visits for participants. A schematic diagram is highly recommended (see Figure) [page 5-11,See detail in Figure. Content for the schedule of enrolment, interventions, and assessments]
Sample size	14	Estimated number of participants needed to achieve study objectives and how it was determined, including clinical and statistical assumptions supporting any sample size calculations [page 12]
Recruitment	15	Strategies for achieving adequate participant enrolment to reach target sample size [page 5]

Methods: Assignment of interventions (for controlled trials)

Allocation:		
Sequence generation	16a	Method of generating the allocation sequence (eg, computer-generated random numbers), and list of any factors for stratification. To reduce predictability of a random sequence, details of any planned restriction (eg, blocking) should be provided in a separate document that is unavailable to those who enrol participants or assign interventions **[page 6]**
Allocation concealment mechanism	16b	Mechanism of implementing the allocation sequence (eg, central telephone; sequentially numbered, opaque, sealed envelopes), describing any steps to conceal the sequence until interventions are assigned **[page 6]**
Implementation	16c	Who will generate the allocation sequence, who will enrol participants, and who will assign participants to interventions **[page 12]**
Blinding (masking)	17a	Who will be blinded after assignment to interventions (eg, trial participants, care providers, outcome assessors, data analysts), and how **[page 12]**
	17b	If blinded, circumstances under which unblinding is permissible, and procedure for revealing a participant's allocated intervention during the trial **[page 11]**
**Methods: Data collection, management, and analysis**		
Data collection methods	18a	Plans for assessment and collection of outcome, baseline, and other trial data, including any related processes to promote data quality (eg, duplicate measurements, training of assessors) and a description of study instruments (eg, questionnaires, laboratory tests) along with their reliability and validity, if known. Reference to where data collection forms can be found, if not in the protocol **[page 12]**
	18b	Plans to promote participant retention and complete follow-up, including list of any outcome data to be collected for participants who discontinue or deviate from intervention protocols **[pages 12-13]**
Data management	19	Plans for data entry, coding, security, and storage, including any related processes to promote data quality (eg, double data entry; range checks for data values). Reference to where details of data management procedures can be found, if not in the protocol **[page 12]**
Statistical methods	20a	Statistical methods for analysing primary and secondary outcomes. Reference to where other details of the statistical analysis plan can be found, if not in the protocol **[pages 12-13]**
	20b	Methods for any additional analyses (eg, subgroup and adjusted analyses) **[pages 12-13]**

	20c	Definition of analysis population relating to protocol non-adherence (eg, as randomised analysis), and any statistical methods to handle missing data (eg, multiple imputation) **[pages 12-13]**
**Methods: Monitoring**		
Data monitoring	21a	Composition of data monitoring committee (DMC); summary of its role and reporting structure; statement of whether it is independent from the sponsor and competing interests; and reference to where further details about its charter can be found, if not in the protocol. Alternatively, an explanation of why a DMC is not needed **[page 12]**
	21b	Description of any interim analyses and stopping guidelines, including who will have access to these interim results and make the final decision to terminate the trial **[page 11]**
Harms	22	Plans for collecting, assessing, reporting, and managing solicited and spontaneously reported adverse events and other unintended effects of trial interventions or trial conduct **[page 11]**
Auditing	23	Frequency and procedures for auditing trial conduct, if any, and whether the process will be independent from investigators and the sponsor **[page 11]**
**Ethics and dissemination**		
Research ethics approval	24	Plans for seeking research ethics committee/institutional review board (REC/IRB) approval **[pages 2,6]**
Protocol amendments	25	Plans for communicating important protocol modifications (eg, changes to eligibility criteria, outcomes, analyses) to relevant parties (eg, investigators, REC/IRBs, trial participants, trial registries, journals, regulators) **[page 11]**
Consent or assent	26a	Who will obtain informed consent or assent from potential trial participants or authorised surrogates, and how (see Item 32) **[page 6]**
	26b	Additional consent provisions for collection and use of participant data and biological specimens in ancillary studies, if applicable **[page 6]**
Confidentiality	27	How personal information about potential and enrolled participants will be collected, shared, and maintained in order to protect confidentiality before, during, and after the trial **[page 6]**
Declaration of interests	28	Financial and other competing interests for principal investigators for the overall trial and each study site **[page 14]**
Access to data	29	Statement of who will have access to the final trial dataset, and disclosure of contractual agreements that limit such access for investigators **[page 16]**

For peer review only

Ancillary and post-trial care	30	Provisions, if any, for ancillary and post-trial care, and for compensation to those who suffer harm from trial participation **[page 11]**
Dissemination policy	31a	Plans for investigators and sponsor to communicate trial results to participants, healthcare professionals, the public, and other relevant groups (eg, via publication, reporting in results databases, or other data sharing arrangements), including any publication restrictions **[pages 2-3]**
	31b	Authorship eligibility guidelines and any intended use of professional writers **[page 15]**
	31c	Plans, if any, for granting public access to the full protocol, participant-level dataset, and statistical code **[page 16]**
**Appendices**		
Informed consent materials	32	Model consent form and other related documentation given to participants and authorised surrogates **[pages 5-6]**
Biological specimens	33	Plans for collection, laboratory evaluation, and storage of biological specimens for genetic or molecular analysis in the current trial and for future use in ancillary studies, if applicable **[page 11]**

*It is strongly recommended that this checklist be read in conjunction with the SPIRIT 2013 Explanation & Elaboration for important clarification on the items. Amendments to the protocol should be tracked and dated. The SPIRIT checklist is copyrighted by the SPIRIT Group under the Creative Commons "Attribution-NonCommercial-NoDerivs 3.0 Unported" license.

Figure. Content for the schedule of enrolment, interventions, and assessments

	STUDY PERIOD				
	Enrolment	Allocation	Post-allocation		Close-out
**TIMEPOINT****		0	*4 weeks*	*8 weeks*	*12weeks*
**ENROLMENT:**					
**Eligibility screen**	X				
**Informed consent**	X				
**Baseline assessment**	X				
**Allocation**		X			
**INTERVENTIONS:**					
**IMR group**			◆———	———◆	
**CR group**			◆———	———◆	
**ASSESSMENTS:**					
**Demographic data**	X	X			
MBI		X	X	X	X
**NIHSS**		X	X	X	X
**FMA**		X	X	X	X
**MMSE & MoCA**		X	X	X	X
**SDS & HAMD**		X	X	X	X
**AEs**			X	X	
**blood routine test, urine routine test, feces routine test, kidney function test, liver function test**		X		X	

For peer review only

Figure 1.Route diagram of study design.

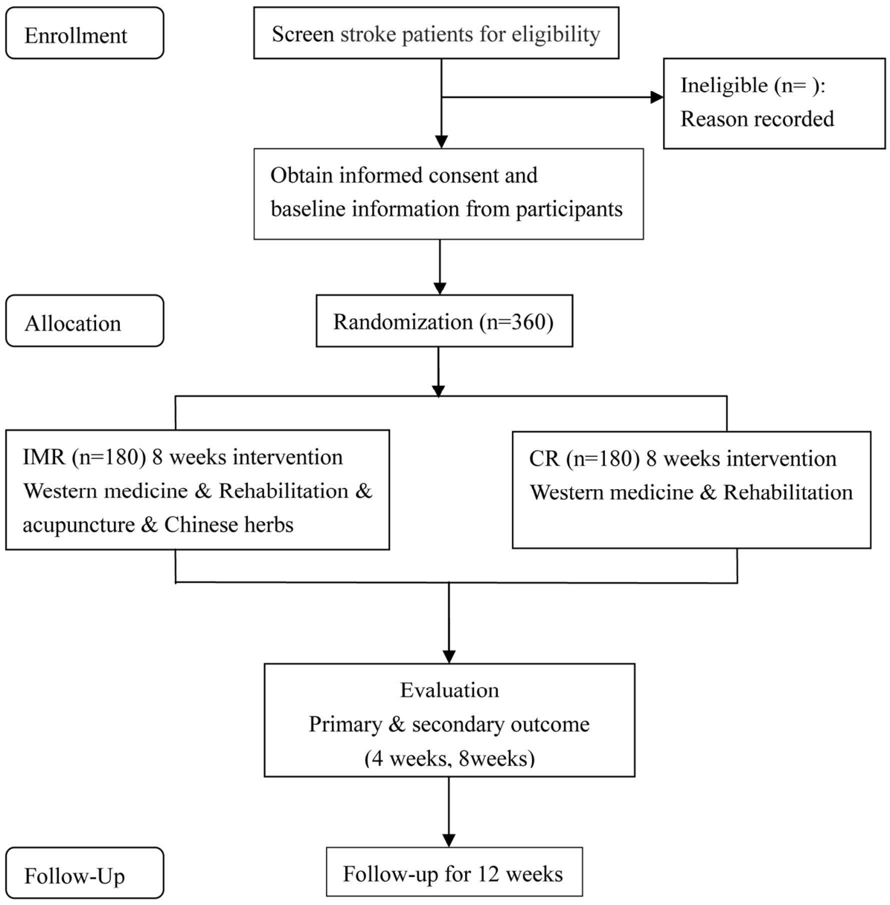

161x176mm (300 x 300 DPI)
