# Supplementary material for: Integrative medicine for subacute stroke rehabilitation: a study protocol for a multicentre, randomised, controlled trial
Source: BMJ Open. 2014 Dec 4;4(12):e007080. doi: 10.1136/bmjopen-2014-007080 (PMC4256536; doi:10.1136/bmjopen-2014-007080)
Supplement: Reviewer comments [file bmjopen-2014-007080.reviewer_comments.pdf]

PEER REVIEW HISTORY

BMJ Open publishes all reviews undertaken for accepted manuscripts. Reviewers are asked to complete a checklist review form (**http://bmjopen.bmj.com/site/about/resources/checklist.pdf**) and are provided with free text boxes to elaborate on their assessment. These free text comments are reproduced below.

ARTICLE DETAILS

**TITLE (PROVISIONAL)**	Integrative Medicine for Sub-acute Stroke Rehabilitation: Study Protocol for a Multicenter, Randomized, Controlled Trial
**AUTHORS**	Fang, Jianqiao; Chen, Lifang; Chen, Luni; Wang, Chao; Keeler, Crystal; Ma, Ruijie; Xu, Shouyu; Shen, Laihua; Bao, Yehua; Ji, Conghua

This article was not externally reviewed at BMJ Open. Protocols that have been independently assessed prior to submission to BMJ Open are usually fast-tracked to publication on the grounds that further substantial changes will not be possible. This independent assessment will usually be external, independent review for both a substantial grant award from a non-commercial or government funder and ethics approval.
